# Heterodimer formed by ROC8 and ROC5 modulates leaf rolling in rice

**DOI:** 10.1111/pbi.13690

**Published:** 2021-09-08

**Authors:** Yang Xu, Weiyi Kong, Fangquan Wang, Jun Wang, Yajun Tao, Wenqi Li, Zhihui Chen, Fangjun Fan, Yanjie Jiang, Qian‐Hao Zhu, Jie Yang

**Affiliations:** ^1^ Institute of Food Crops Jiangsu Academy of Agricultural Sciences Nanjing China; ^2^ Jiangsu Co‐Innovation Center for Modern Production Technology of Grain Crops Yangzhou University Yangzhou China; ^3^ Provincial Key Laboratory of Agrobiology Jiangsu Academy of Agricultural Sciences Nanjing China; ^4^ College of Grassland Science Nanjing Agricultural University Nanjing China; ^5^ Agriculture and Food CSIRO Canberra ACT Australia

**Keywords:** rice, leaf rolling, bulliform cell, transgenic plant, ROC transcription factor, protein interaction, Crispr/Cas9

## Abstract

Moderately rolled leaf is one of the target traits of the ideal plant architecture in rice breeding. Many genes, including homeodomain leucine zipper IV transcription factors *ROC5* and *ROC8*, regulating rice leaf rolling have been cloned and functionally analysed. However, the molecular mechanism by which these genes modulate leaf‐rolling remains largely elusive. In this study, we demonstrated the transcription activation activity of both ROC8 and ROC5. Overexpressing *ROC8* caused adaxially rolled leaves due to decreased number and size of bulliform cells, whereas knockout of *ROC8* induced abaxially rolled leaves due to increased number and size of bulliform cells. ROC8 and ROC5 each could form homodimer, but ROC8 interacted preferably with ROC5 to forms a heterodimer. Importantly, we showed that the ROC8‐ROC5 heterodimer rather than the homodimer of ROC8 or ROC5 was functional as neither overexpressing *ROC8* in the *ROC5* mutant nor overexpressing *ROC5* in the *ROC8*‐knockout line could rescue the mutant phenotype. This was further partially supported by the identification of a large number of common differentially expressed genes in single and double mutants of *roc8* and *roc5*. *ROC8* and *ROC5* were functionally additive as the phenotype of abaxially rolled leaves was stronger in the *roc5roc8* double mutant than in their single mutants. Our results provide evidence for the role of dimerization of ROC members in regulating leaf rolling of rice.

## Introduction

Appropriate leaf morphology is an important target trait in breeding of rice (*Oryza sativa* L.), the staple food for more than half of the world's population. As an ideal trait to achieve the goal of higher yield, moderate leaf rolling in rice is helpful to delay leaf senescence, keep leaf erect to increase the light‐receiving space, and enhance the light quantity and intensity at the base of rice plants in the field (Price *et al*., 1997; Xu *et al*., 2018; Zhang *et al*., 2009). Isolation of the genes controlling leaf rolling will be beneficial for achieving the desired rice plant architecture through conventional and modern molecular breeding approaches. Several genes regulating leaf rolling have been identified and characterized in rice. Some were known to affect the development of leaf abaxial cells (Zhang *et al*., 2009), the cell wall formation, epidermis integrity, and water homeostasis (Li *et al*., 2017). But most of the genes, such as *NRL1* (Hu *et al*., 2010), *ACL1*/*ACL2* (Li *et al*., 2010), *ROC5* (Zou *et al*., 2011), *RL14* (Fang *et al*., 2012), *SRL1* (Xiang *et al*., 2012), and *OsZHD1* (Xu *et al*., 2014) were reported to control leaf rolling by regulating the number and/or the size of the bulliform cells, which are special types of cells existing specifically on the adaxial side of leaves in gramineous plants. Despite these progresses, our understanding on the molecular mechanism underpinning leaf rolling in rice still remains fragmented.

Plant‐specific homeodomain leucine zipper IV (HD‐Zip IV) genes encode a class of homeobox transcription factors (Elhiti and Stasolla, 2009), which contain two conserved domains, homeobox (HD) and START‐ArGLABRA2‐like (START). HD‐Zip IV, or HD‐GL2, transcription factors have diverse functions in plant development. They have been shown to be involved in trichome development (Vernoud *et al*., 2009), cuticular wax biosynthesis (Wang *et al*., 2018), and plant morphogenesis (Sun *et al*., 2020; Zou *et al*., 2011). The rice genome contains nine HD‐GL2 members, *rice outermost cell‐specific1* (*ROC1*) to *ROC9* (Ito *et al*., 2003). Of these, *ROC5* and *ROC8* have been reported to play important roles in controlling leaf rolling by affecting the formation and development of bulliform cells (Sun *et al*., 2020; Zou *et al*., 2011). *ROC5* was identified based on characterization of a T‐DNA insertion mutant (*outcurved leaf1* or *oul1*) showing abaxial leaf rolling thanks to increased number and size of bulliform cells. Overexpressing *ROC5* resulted in leaf rolling in the opposite direction, that is, adaxial rolling, due to decreased number and size of bulliform cells (Zou *et al*., 2011). Map‐based cloning identified *ROC8,* the gene responsible for the mutant phenotype of adaxially rolled leaves in *crm1‐D* caused by reduced size of bulliform cells (Sun *et al*., 2020). The expression level of *ROC8* is similar between *crm1‐D* and its wild type, Nipponbare, but the protein level of ROC8 is significantly higher in the mutant than in Nipponbare, probably due to lack of a 30‐bp sequence in the 3’ UTR of *ROC8* in *crm1‐D* that acts as a translational repressor (Sun *et al*., 2020). This finding implies the importance of ROC proteins rather than *ROC* transcripts themselves in regulating leaf rolling. However, it is currently unknown how ROC proteins achieve their role in modulating development of bulliform cells and consequently leaf rolling.

We herein reported that overexpression and knockout of *ROC8* resulted in the same mutant phenotypes as those observed when overexpressing and silencing *ROC5*, respectively. Analyses of protein interactions demonstrated that both ROC8 and ROC5 could individually form homodimers but they tended to form heterodimers, which seem to be the functional protein complex involved in regulating expansion and division of bulliform cells, and finally leaf rolling.

## Results

### Both ROC8 and ROC5 have transcriptional activation activity

**Figure 1:**
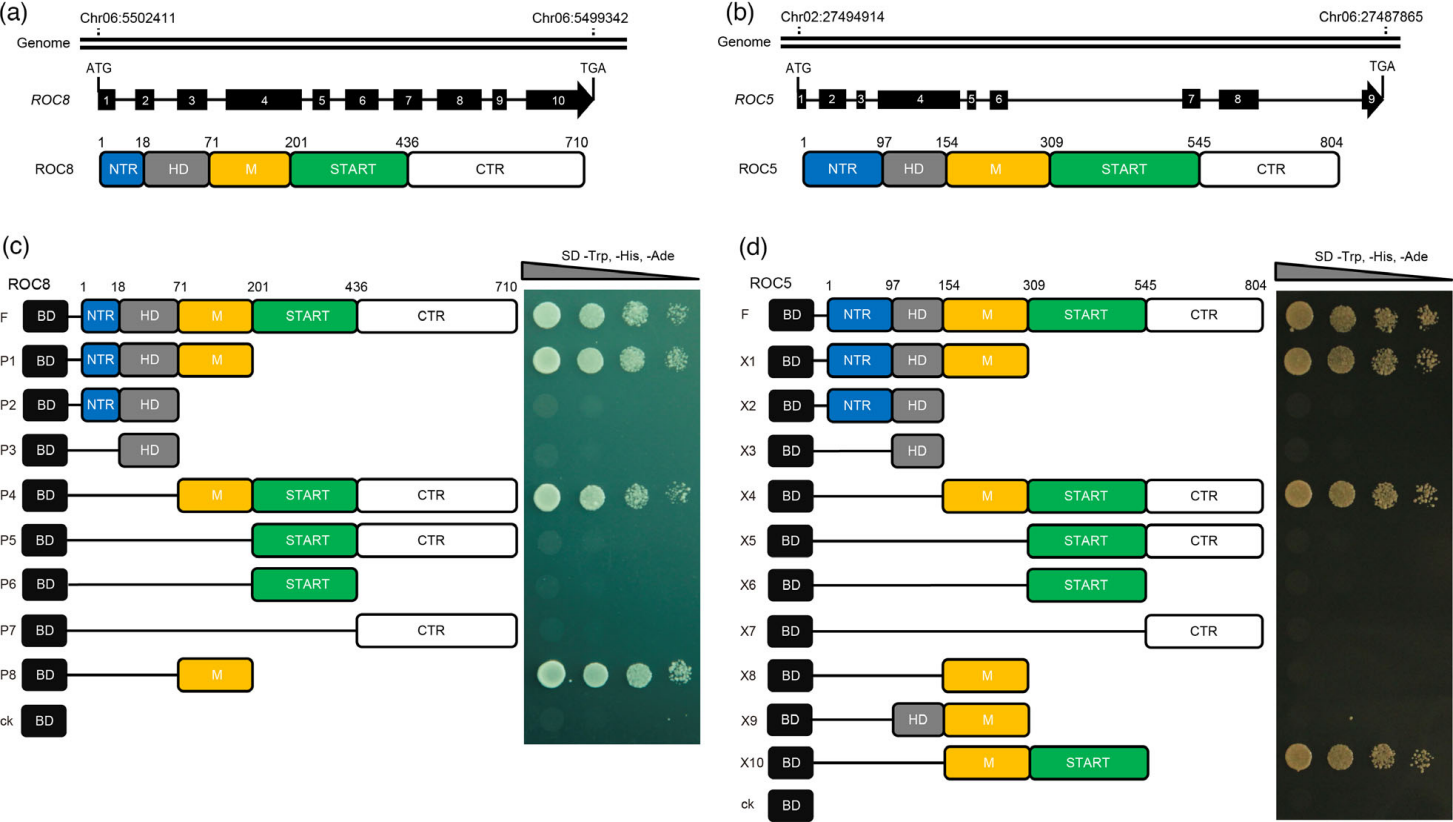
ROC8 and ROC5 both have transcriptional activation activity. (a, b) Schematic diagrams of *ROC8* and its protein (a), and *ROC5* and its protein (b). Filled black boxes with Arabic numerals and bold lines represent exon and intron, respectively (middle panel). Numbers indicate the amino acid position (bottom panel). NTR, N‐terminal region; HD, Homeodomain; M, Middle region; START, START‐ArGLABRA2‐like domain; CTR, C‐terminal region. (c, d) Transcription activation activity assay of ROC8 (c) and ROC5 (d) in yeast system. Numbers indicate the amino acid position. P1 to P8 indicate a series of truncated ROC8 proteins fused with the BD. X1 to X10 indicate a series of truncated ROC5 proteins fused with the BD. The pGBKT7 vector with only the BD was used as a negative control.

*ROC8* and *ROC5*, two transcription factors of the HD Zip IV family, regulate the size of bulliform cells and consequently leaf rolling in rice (Sun *et al*., 2020; Zou *et al*., 2011). We found that *ROC8*, a ubiquitously expressed nuclear gene (Figure S1a,b), and *ROC5*, can be found in the third leaf of different rice varieties (Figure S1c,d). ROC8 and ROC5 have the same protein structure, containing the conserved HD domain [amino acids (aa) 18‐70 for ROC8 and 97‐153 aa for ROC5] and START domain (201‐435 aa for ROC8 and 309‐544 aa for ROC5), N‐terminal region (NTR, 1‐17 aa for ROC8 and 1‐96 aa for ROC5), Middle region (M, 71‐200 aa for ROC8 and 154‐308 aa for ROC5), and C‐terminal region (CTR, 436‐710 aa for ROC8 and 545‐804 aa for ROC5) (Figure 1a,b).

To determine whether ROC8 has transcriptional activation activity, a series of protein truncations fused with the GAL4 DNA‐binding domain (BD) were constructed and subsequently analysed in yeast. The results showed that the yeast strains transformed with the region comprising amino acids 1‐710 (F), 71‐710 (P4), or 71‐200 (P8) grew well on the SD/‐Trp/‐His/‐Ade medium, suggesting that the full‐length ROC8 and the M region of ROC8 had transcriptional activation activity (Figure 1c). We also analysed the transcriptional activation activity of ROC5, using different protein truncations containing one or more domains/regions. Like ROC8, ROC5 also had transcriptional activation activity, but required not only the M region but additional regions, including NTR+HD (X1), START (X10), or START+CTR (X4) (Figure 1d).

### Both ROC8 and ROC5 are able to form homodimers

**Figure 2:**
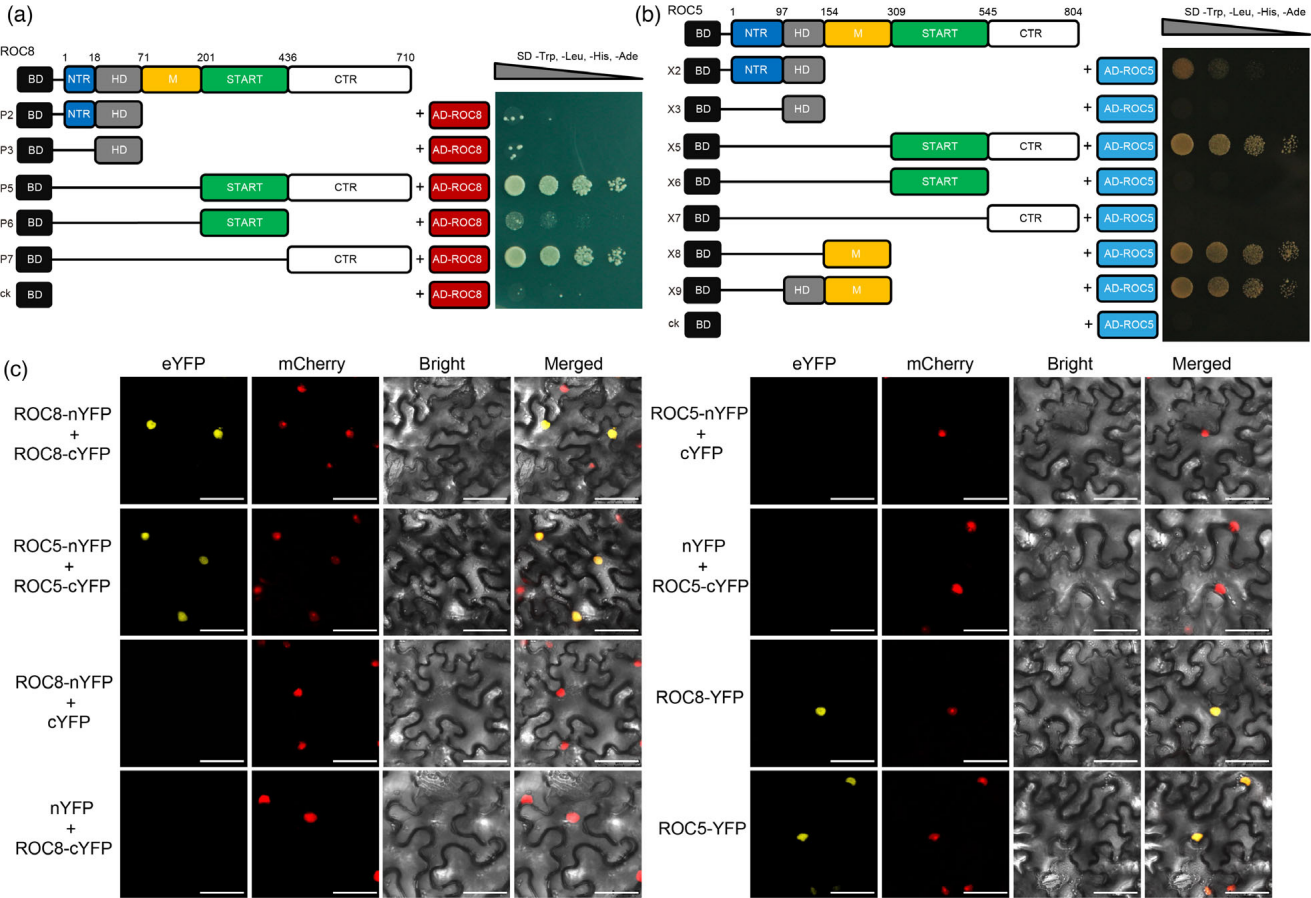
ROC8 and ROC5 can form homodimers. (a, b) Homodimeric interaction of ROC8 (a) and ROC5 (b) in the yeast system. Numbers indicate the amino acid position. P2, P3, P5, P6, and P7 indicate a series of truncated ROC8 proteins without transcriptional activation activity fused with the BD. X2, X3, X5, X6, X7, X8, and X9 indicate a series of truncated ROC5 proteins without transcriptional activation activity fused with the BD. The pGBKT7 vector with only the BD was used as a negative control (ck). (c) BiFC assay. nYFP and cYFP stand for the N terminus and C terminus of eYFP, respectively. eYFP, enhanced yellow fluorescence protein; mCherry, fluorescence of nuclear marker D53‐mCherry fusion protein; Bright, bright‐field; Merged, merged image of eYFP, mCherry, and Bright. Bars:100 μm.

Plant HD‐ZIP IV family members may function via homodimers in planta (Ito *et al*., 2003). To evaluate the homodimeric interaction of ROC8 or ROC5, the protein truncations identified as without transcriptional activation activity (Figure 1c,d; Figure S2) were co‐transformed with AD‐ROC8 or AD‐ROC5. For ROC8, the yeast strains transformed with the region comprising amino acids 201‐710 (P5) and 436‐710 (P7) grew well on the SD medium (‐LTHA), suggesting that CTR was required for the homodimeric interaction (Figure 2a). For ROC5, START+CTR (X5), M region (X8), or HD+M (X9) region could interact with the full‐length ROC5 to form homodimers (Figure 2b). To determine whether full‐length ROC8 or ROC5 protein could form homodimers in plant cells, we employed the bimolecular fluorescence complementation (BiFC) assay. The results showed that when ROC8‐nYFP was co‐expressed with ROC8‐cYFP in tobacco leaves, the eYFP signal was very strong in the nucleus, whereas no signal was detected in the negative controls. Similar results were observed for the co‐expression of ROC5‐nYFP and ROC5‐cYFP (Figure 2c). Meanwhile, both ROC8‐YFP and ROC5‐YFP showed florescence signal in nucleus, consistent with the result of the subcellular localization experiment of ROC8 (Figure S1b) and previous results on the subcellular localization of ROC5 (Zou *et al*., 2011). These results, together, demonstrate that both ROC8 and ROC5 are capable of forming homodimers.

### Overexpression of *ROC8* causes adaxially rolled leaves and knockout of *ROC8* induces abaxially rolled leaves

To elucidate the role of *ROC8* in rice morphogenesis, we overexpressed the full‐length *ROC8* driven by the 35S promoter in *japonica* rice variety Kitaake. In total, ten independent positive transgenic lines were obtained, and among them, seven lines exhibited the phenotype of adaxial leaf rolling. Three independent lines, OE‐1, OE‐2, and OE‐3, were selected for analysis in detail. Compared with the upright and flat leaf blades in Kitaake, the leaves of these lines were adaxially rolled (Figure S3a). Leaf adaxial‐rolling indexes were measured. While Kitaake had a leaf adaxial‐rolling index of 0 (unrolled and flat leaves), the leaf adaxial‐rolling indexes of the three lines were all greater than 82 (Figure S3b). The qRT‐PCR analysis demonstrated that the expression level of *ROC8* in these transformants was up‐regulated in varying degrees, indicating that *ROC8* was successfully overexpressed (Figure S3c) and seemed to be positively correlated with the leaf adaxial‐rolling index.

Furthermore, we created *ROC8*‐knokout mutants by Crispr/Cas9‐mediated gene editing technology. We identified ten mutated transgenics, and all exhibited abaxial leaf rolling. Three independent lines, crispr8‐6, crispr8‐7, and crispr8‐8, were selected for analysis in detail. All three mutants showed a leaf abaxial‐rolling index higher than Kitaake (Figure S3d–f), although they seemed to have a normal expression of *ROC8* (Figure S4), suggesting that ROC8 is mutated in all three mutants due to the 1‐2 bp indels (causing frame‐shift) induced by gene editing (Figure S3d) and that the ROC8 protein, rather than the *ROC8* transcript, is important for its functionality.

To dissect the cellular differences responsible for the leaf rolling in *ROC8* overexpression (adaxially rolled) and knockout (abaxially rolled) transgenic lines, we compared their bulliform cells abutting the large vascular and those between the two small vascular bundles, using leaf cross sections. We found that, compared with Kitaake, the overexpression lines contained less and smaller bulliform cells, while in contrast, the knockout lines contained more and bigger bulliform cells (Figure S3g–i). These observations suggest that ROC8 is a negative regulator of not only the number but also the size of bulliform cells and that both the number and size of bulliform cells play a role in leaf rolling.

### ROC8 physically interacts with ROC5

Given that the expression of *ROC8* overlapped with that of *ROC5* (Figure S1c,d), and ROC8 has a similar biological function as ROC5 (Zou *et al*., 2011) in regulating leaf rolling, it was of our interest to know their relationship. We first analysed the expression level of *ROC5* in *ROC8* overexpression and knockout plants, and the expression level of *ROC8* in the T‐DNA insertion mutant of *ROC5*, *oul1*. We found no difference of *ROC5* expression between the *ROC8* mutants and the wild‐type Kitaake (Figure S5a,b). Similarly, the expression level of *ROC8* in the *oul1* mutant was comparable to that in the wild‐type Nipponbare (Figure S5c). These findings indicate that overexpression/knockout of *ROC8* does not affect the transcription level of *ROC5*, and that loss‐of‐function of *ROC5* does not affect the expression of *ROC8*.

**Figure 3:**
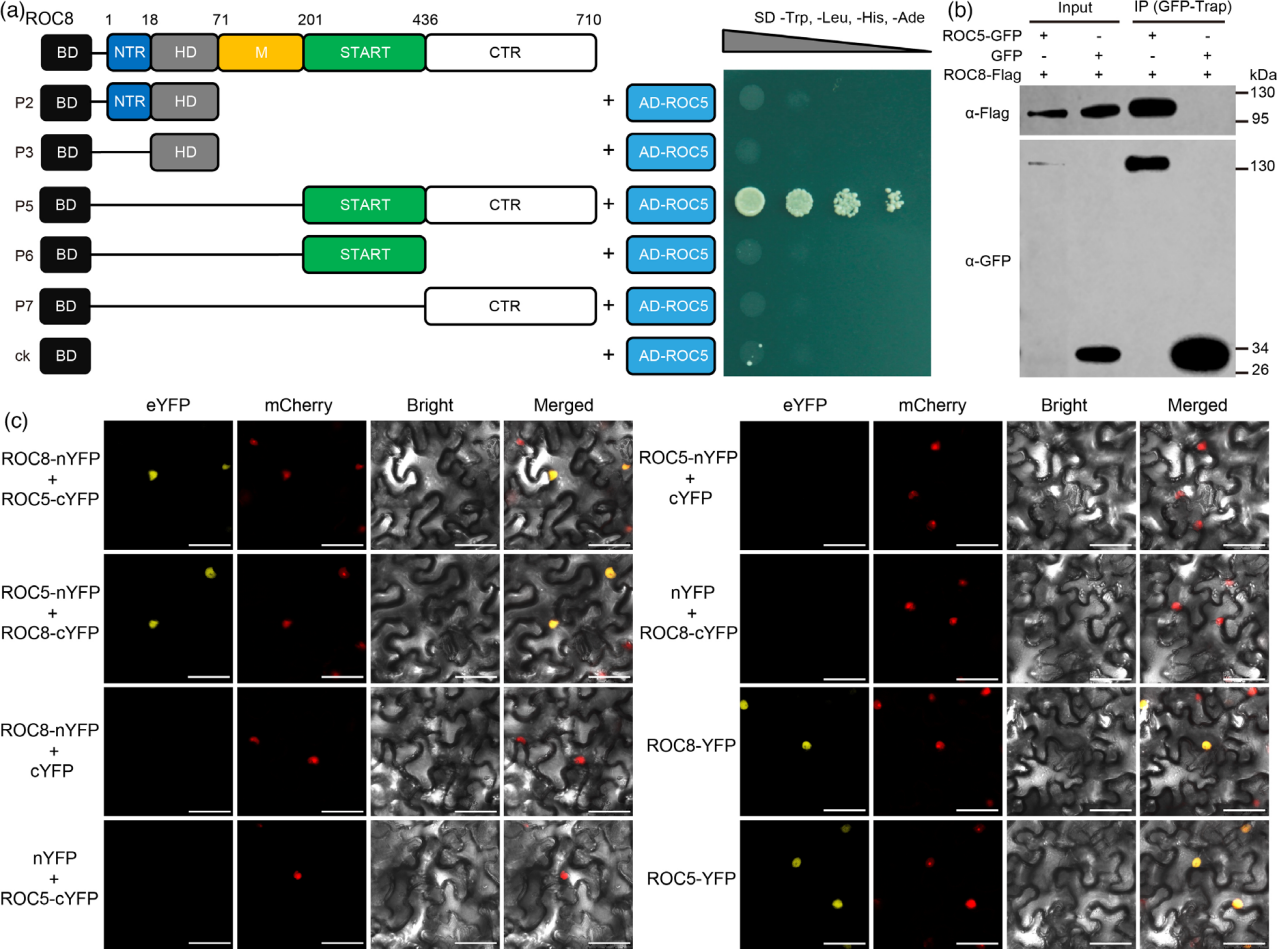
ROC8 physically interacts with ROC5. (a) Yeast two‐hybrid assay. Numbers indicate the amino acid position. P2, P3, P5, P6, and P7 indicate a series of ROC8 protein truncations without transcriptional activation activity fused with the BD. The pGBKT7 vector with only the BD co‐transformed with the ROC5 fused with the AD was used as a negative control. (b) Co‐IP assay showing that ROC8‐Flag can be coimmunoprecipitated in the extract of rice protoplasts with GFP‐Trap. The left two lanes show the protein immunoblots of input controls with anti‐Flag or anti‐GFP antibody. The right two lanes show the results of Co‐IP. (c) BiFC assay. nYFP and cYFP stand for the N terminus and C terminus of eYFP, respectively. eYFP, enhanced yellow fluorescence protein; mCherry, fluorescence of nuclear marker D53‐mCherry fusion protein; Bright, bright‐field; Merged, merged image of eYFP, mCherry, and Bright. Bars:100 μm.

Then, we performed the yeast two‐hybrid assay to test whether ROC8 interacts with ROC5. We found that the strain co‐transformed with the BD construct containing the region of START+CTR (P5) and the GAL4 activation domain (AD) construct containing ROC5 could grow in the SD medium (‐LTHA), but the strains co‐transformed with other ROC8 regions did not survive (Figure 3a), indicating that the ROC8 region containing the START domain and CTR can interact with ROC5. We also tested the interactions between different ROC5 protein truncations and ROC8 and found that the ROC5 region containing START+CTR (309‐804 aa, X5) or the M region (154‐308 aa, X8 and X9) could interact with ROC8 (Figure S6). To determine the interaction between ROC8 and ROC5 in plant cells, we employed the co‐immunoprecipitation (Co‐IP) and BiFC assays. The results showed that the full‐length ROC8 could interact with the full‐length ROC5 in rice protoplasts (Figure 3b) and in the cell nuclei of tobacco leaves (Figure 3c). Together, these results suggest that ROC8 can physically interact with ROC5, that is, ROC8 and ROC5 can form heterodimer.

### Heterodimerization between ROC8 and ROC5 competes with homodimerization of ROC8 or ROC5

**Figure 4:**
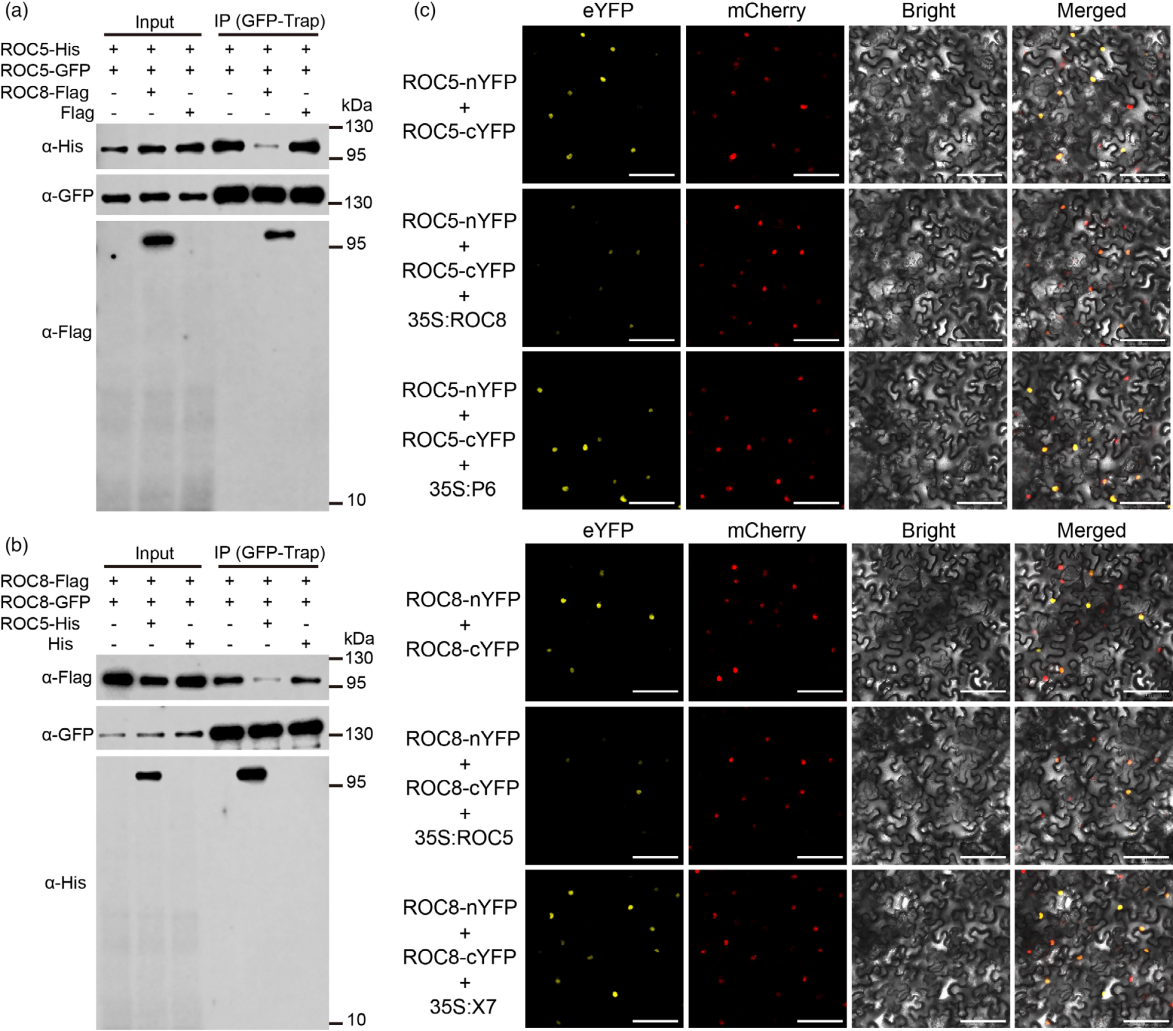
Competitive relationship between the ROC8‐ROC5 heterodimerization and homodimerization of ROC8 or ROC5. (a) Co‐IP assay showing the competition of ROC8 and ROC5 heterodimerization and ROC5 homodimerization. The left three lanes show the protein immunoblots of input controls with anti‐His, anti‐GFP, or anti‐Flag antibodies. The right three lanes show the results of Co‐IP. The GFP‐Trap was used for the coimmunoprecipitation. (b) Co‐IP assay showing the competition of ROC8 and ROC5 heterodimerization and ROC8 homodimerization. The left three lanes show the protein immunoblots of input controls with anti‐Flag, anti‐GFP, or anti‐His antibodies. The right three lanes show the results of Co‐IP. The GFP‐Trap was used for the coimmunoprecipitation. (c) BiFC assay. The P6 truncated ROC8 and the X7 truncated ROC5 were used as the negative controls. nYFP and cYFP stand for the N terminus and C terminus of eYFP, respectively. eYFP, enhanced yellow fluorescence protein; mCherry, fluorescence of nuclear marker D53‐mCherry fusion protein; Bright, bright‐field; Merged, merged image of eYFP, mCherry and Bright. Bars:100 μm.

Given that the same regions were involved in the interaction between ROC8 and ROC5 (START+CTR for ROC8, START+CTR or M region for ROC5, Figure 3a, Figure S6), and in the homodimerization of ROC8 (START+CTR, Figure 2a) or ROC5 (START+CTR or M region, Figure 2b), we speculated that heterodimerization between ROC8 and ROC5 may be interfered by homodimerization of ROC8 or ROC5. We performed Co‐IP assay to test this possibility and found that ROC5‐His could be co‐immunoprecipitated by ROC5‐GFP in the extract of rice protoplasts (Figure 4a) with GFP‐Trap, consistent with the result that ROC5 was capable of forming homodimers (Figure 2b,c). While when ROC8‐Flag, but not Flag itself, was co‐transfected with ROC5‐His and ROC5‐GFP, the ROC5‐His co‐immunoprecipitated by ROC5‐GFP decreased tremendously (Figure 4a), indicating that homodimerization of ROC5 decreases in the presence of ROC8. Similarly, ROC8‐Flag could be co‐immunoprecipitated by ROC8‐GFP in the extract of rice protoplasts (Figure 4b) with GFP‐Trap, consistent with the result that ROC8 was capable of forming homodimers (Figure 2a,c). While when ROC5‐His, but not His itself, was co‐transfected with ROC8‐Flag and ROC8‐GFP, homodimerization of ROC8, that is, co‐immunoprecipitation of ROC8‐Flag and ROC8‐GFP, dramatically decreased (Figure 4b), indicating that heterodimerization between ROC8 and ROC5 competes with homodimerization of ROC8.

Furthermore, we used BiFC assay to investigate competition between homodimerization and heterodimerization of ROC8 and ROC5. When ROC5‐nYFP was co‐expressed with ROC5‐cYFP in tobacco leaves (as a control), the eYFP signal was very strong in the nucleus. However, in the presence of ROC8, the interaction between ROC5‐nYFP and ROC5‐cYFP became weaker as indicated by the weaker eYFP signal (Figure 4c, compares the middle image to the top one of the upper panel). When a truncated ROC8 (P6), which could not interact with ROC5 (Figure 3a), was co‐expressed with ROC5‐nYFP and ROC5‐cYFP, the eYFP signal was the same strength as in the control (Figure 4c). Similarly, when ROC5 was co‐expressed with ROC8‐nYFP and ROC8‐cYFP, the eYFP signal also became weaker compared to the co‐expression of ROC8‐nYFP and ROC8‐cYFP, and a truncated ROC5 (X7) that could not interact with ROC8 had no effect on homodimerization of ROC8 (Figure 4c, the lower panel). These results confirmed that the heterodimerization of ROC8 and ROC5 competed with the homodimerization of either ROC8 or ROC5.

### Overexpression of *ROC8* in the *oul1* mutant unable to rescue the mutant phenotype of abaxially rolled leaf

**Figure 5:**
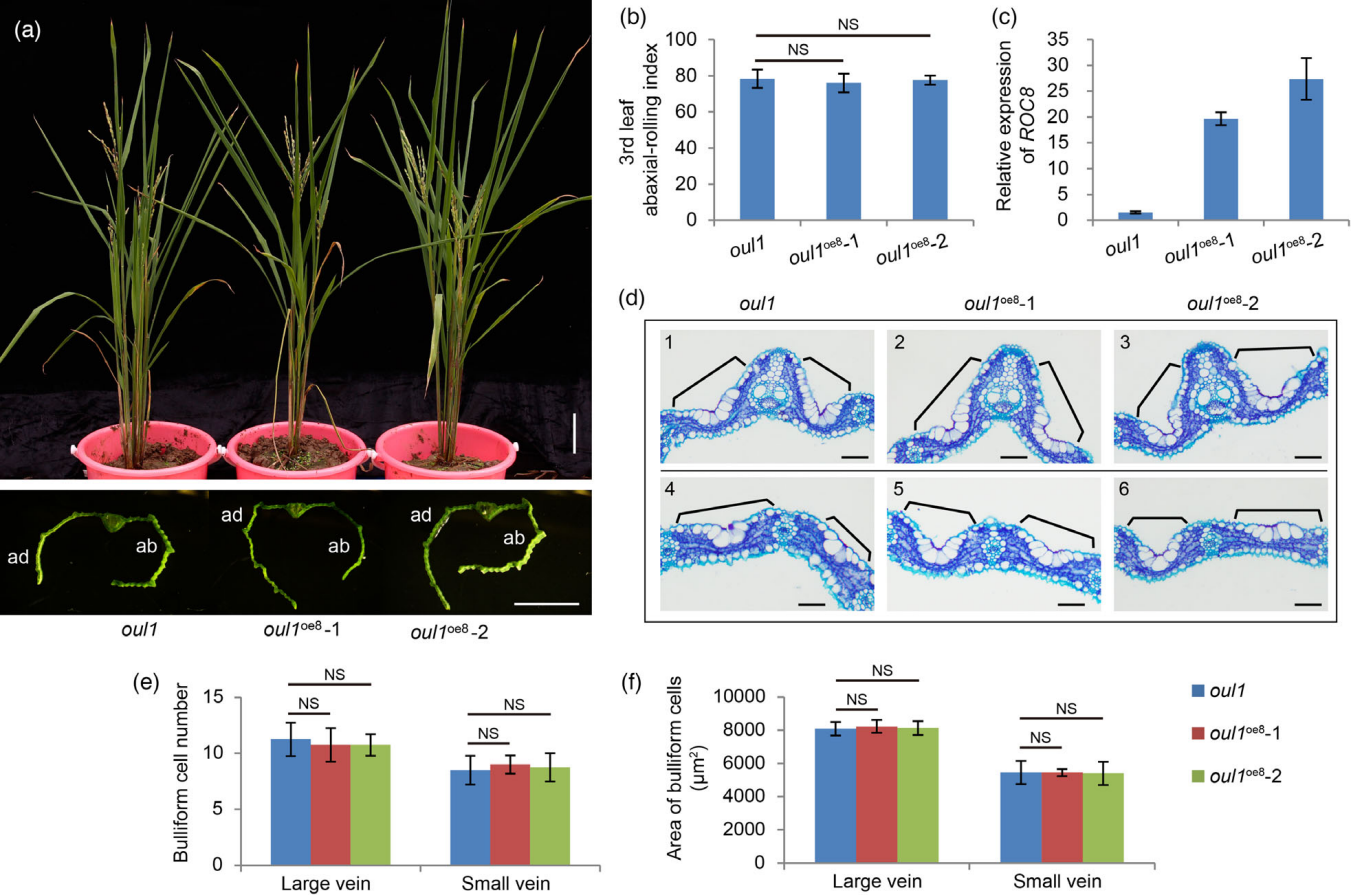
Overexpressing *ROC8* in the *oul1* mutant. (a) Plant stature (top) and transverse leaf sections (bottom) of the *oul1* mutant and two independent lines (*oul1* ^oe8^‐1 and *oul1* ^oe8^‐2) with overexpressed *ROC8* in the *oul1* background at the heading stage. Note that the leaves of the overexpression lines remained abaxially rolled. ab, abaxial; ad, adaxial. Bars: 10 cm (top) and 5 mm (bottom). (b) Leaf abaxial‐rolling index of the third leaf. Data are means ± SD (n = 30). (c) qRT‐PCR analysis of *ROC8* expression. Data are means ± SD (n = 3). (d) Cross sections showing the bulliform cells abutting large vascular (panels 1 to 3) and those between the two small vascular bundles (panels 4 to 6) in the *oul1* mutant and the two *ROC8* overexpression lines. Black bracket lines indicate the bulliform cells. Bars: 50 μm. (e, f) Statistical analysis of the number (e) and area (f) of bulliform cells. Data are means ± SD (n = 4). Statistical analysis was done by the Student's *t*‐test (NS, no significant difference).

To further elucidate the biological function of ROC8 and ROC5 in the regulation of leaf rolling, we overexpressed the *ROC8* gene in the *oul1* mutant and generated five independent T_0_ positive transgenic lines that all exhibited abaxially rolled leaves. Two independent lines, *oul1* ^oe8^‐1 and *oul1* ^oe8^‐2, were selected for detailed phenotyping. Compared to the *oul1* mutant, both *oul1* ^oe8^‐1 and *oul1* ^oe8^‐2 had similar overall plant morphology and abaxial‐rolling indexes (Figure 5a,b), although the expression level of *ROC8* was dramatically higher in the overexpressing lines than in *oul1* (Figure 5c). Furthermore, the morphology, number, and size of the bulliform cells abutting large vascular bundle and those between the two small vascular bundles were also similar between the two overexpressing lines and *oul1* (Figure 5d–f). Therefore, overexpression of *ROC8* in *oul1* could not rescue the mutant phenotype (abaxially rolled leaf) of *oul1*, let alone induce adaxially rolled leaf as observed in the *ROC8* overexpression lines with the Kitaake background (Figure S3a).

### Overexpression of *ROC5* in the line crispr8‐6 unable to rescue the mutant phenotype of abaxially rolled leaf

**Figure 6:**
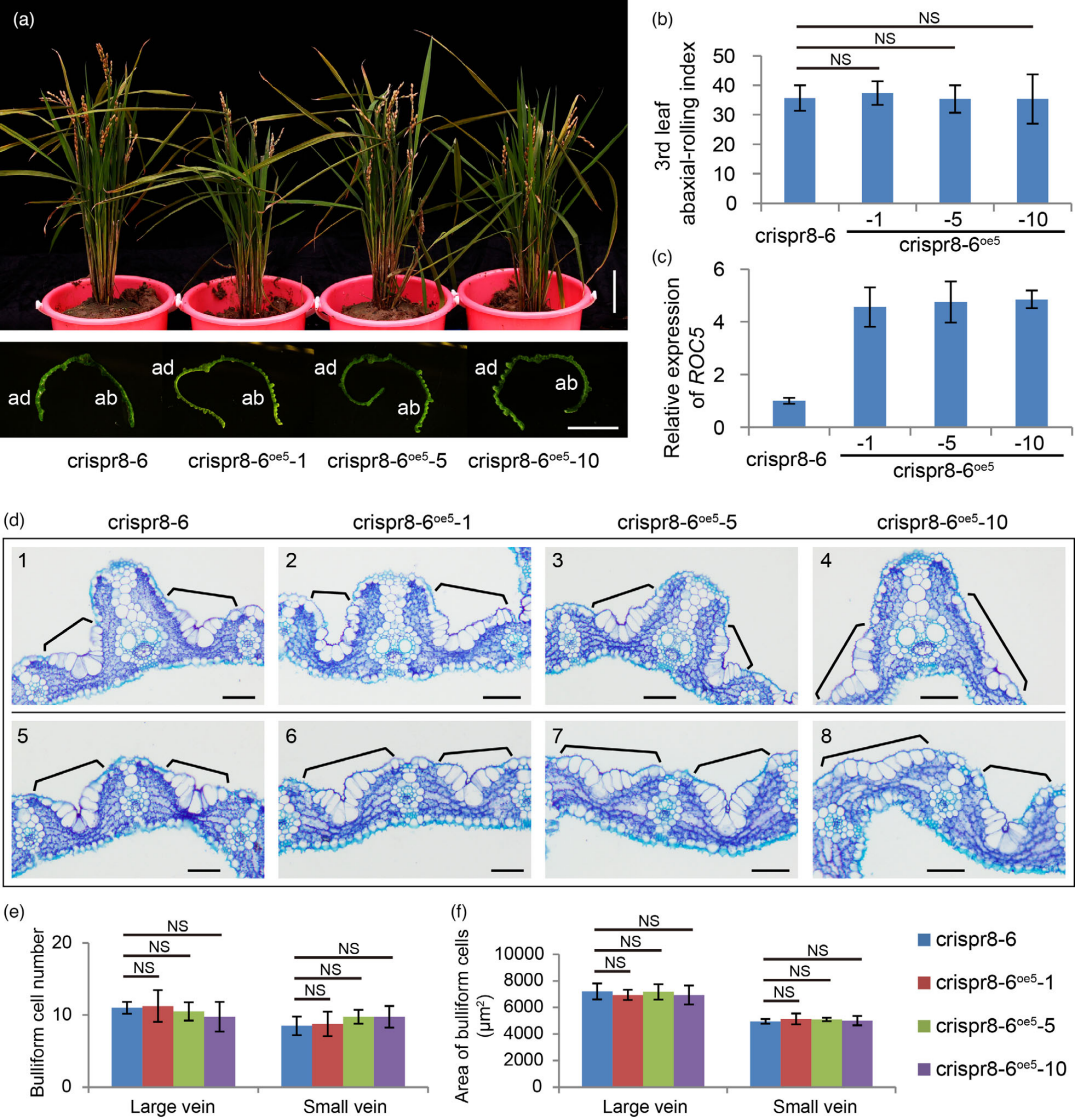
Overexpressing *ROC5* in the *ROC8*‐knockout line crispr8‐6. (a) Plant stature (top) and transverse leaf sections (bottom) of line crispr8‐6 and three independent lines (crispr8‐6^oe5^‐1, crispr8‐6^oe5^‐5, and crispr8‐6^oe5^‐10) with overexpressed *ROC5* in the line crispr8‐6 background at the heading stage. Note that the leaves of overexpression lines remained abaxially rolled. ab, abaxial; ad, adaxial. Bars: 10 cm (top) and 5 mm (bottom). (b) Leaf abaxial‐rolling index of the third leaf. Data are means ± SD (n = 30). (c) qRT‐PCR analysis of *ROC5* expression. Data are means ± SD (n = 3). (d) Cross sections showing the bulliform cells abutting large vascular (panels 1 to 4) and those between the two small vascular bundles (panels 5 to 8) in the line crispr8‐6 and the three *ROC5* overexpression lines. Black bracket lines indicate the bulliform cells. Bars: 50 μm. (e, f) Statistical analysis of the number (e) and area (f) of bulliform cells. Data are means ± SD (n = 4). Statistical analysis was done by the Student's *t*‐test (NS, no significant difference).

We also overexpressed *ROC5* in the *ROC8*‐knockout line crispr8‐6. All ten independent T_0_ positive transgenic lines exhibited abaxially rolled leaves. Three representative independent lines, crispr8‐6^oe5^‐1, crispr8‐6^oe5^‐5, and crispr8‐6^oe5^‐10, are shown in Figure 6a. The abaxial‐rolling indexes of these three lines had no difference from that of crispr8‐6 (Figure 6b), although the *ROC5* mRNA level was obviously higher in the overexpressing lines than in crispr8‐6 (Figure 6c). Moreover, the morphology, number, and size of the bulliform cells abutting large vascular and those between the two small vascular bundles of the overexpressing lines could not be distinguished from those in crispr8‐6 (Figure 6d–f). These results indicate that overexpression of *ROC5* in the line with mutated ROC8 is unable to rescue the mutant phenotype of the abaxially rolled leaf.

### Identification of genes regulated by both ROC5 and ROC8

We further created *roc5roc8* double mutants by Crispr/Cas9‐mediated gene editing (Figure S7a) to investigate the function of ROC5 and ROC8 in rice development, particularly in leaf development. All seven independent positive T_0_ transgenic lines, which were homozygous or biallelic frameshift mutants (Table S1), exhibited severely abaxially rolled leaves as demonstrated by the three representative lines, crispr5&8‐1, crispr5&8‐2, and crispr5&8‐3, shown in Figure S7b. The leaf abaxial‐rolling indexes of the three double knockout mutants were all significantly higher than that of the *roc8* single mutant line crispr8‐6 (Figure S7c), accompanied by varying degrees of increase in bulliform cell numbers and size (Figure S7d–f), suggesting that the role of ROC5 and ROC8 in regulating leaf rolling is additive.

**Figure 7:**
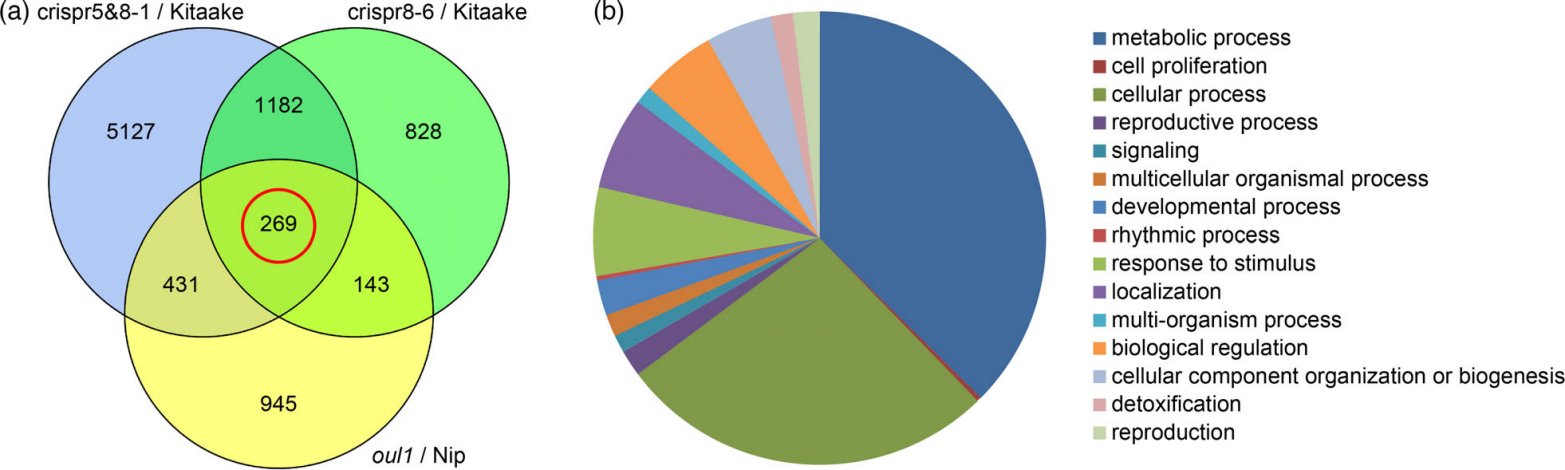
Comparative transcriptome analysis. (a) Venn diagram showing the number of differentially expressed genes (*P* < 0.05) in the double mutant line crispr5&8‐1, the single mutant line crispr8‐6, and the *oul1* mutant compared with their corresponding wild type. (b) Gene Ontology analysis of the differentially expressed genes (n = 269, circled in red in (a)) shared by the three mutants, that is, crispr5&8‐1, crispr8‐6, and *oul1*. Three biological replicates from each line were used in RNA‐seq.

In order to identify the genes regulated by ROC5 and ROC8, we compared the transcriptomes of the double mutant line crispr5&8‐1, the *roc8* single mutant line crispr8‐6, and the *oul1* (*roc5*) mutant. Differentially expressed genes (DEGs) between each mutant and its corresponding wild type were first identified and the DEGs were then compared to identify those shared by the three mutants. Compared with Kitaake, the double mutant crispr5&8‐1 had 3377 and 3632 up‐regulated and down‐regulated genes, respectively (Data S1), and the single mutant crispr8‐6 had 1863 and 559 up‐regulated and down‐regulated genes, respectively (Data S2). Compared to Nipponbare, the *oul1* mutant had 1166 and 622 up‐regulated and down‐regulated genes, respectively (Data S3). Among these DEGs, 269 were common in the three mutants (Figure 7a, Data S4), suggesting that many genes may be directly or indirectly regulated simultaneously by *ROC5* and *ROC8*. Gene Ontology (GO) analysis of these 269 genes revealed that approximately two‐thirds of them were significantly enriched with genes involved in metabolic process and cellular process (Figure 7b).

It has been shown that, besides controlling the formation of bulliform cells, several rolling‐leaf‐related genes were associated with cellulose and lignin biosynthesis, cell wall and vacuole formation, and water stress (Xu *et al*., 2018). We found that, of the 269 DEGs, at least ten might function in these biological and physiological processes (Table S2). Whether these genes are the direct targets of the ROC8‐ROC5 heterodimer in rice leaves remains to be investigated in future studies.

## Discussion

Many genes regulating leaf morphology, including several regulating leaf rolling, have been identified in rice (Xu *et al*., 2018). In this study, we found that ROC5 and ROC8, two members of the HD‐ZIP IV transcription factor family that had been shown to function in regulating rice leaf rolling (Sun *et al*., 2020; Zou *et al*., 2011), possess transcription activation activity (Figure 1c,d). Overexpressing *ROC8* caused adaxially rolled leaves with decreased number and size of bulliform cells in the epidermal layer of rice leaves. Knockout of *ROC8* increased the number and size of bulliform cells and induced abaxially rolled leaves (Figure S3). These results strongly suggest that, like *SRL1* (Xiang *et al*., 2012), *ROC5* (Zou *et al*., 2011), and *ACL1*/*ACL2* (Li *et al*., 2010), *ROC8* plays a significant role in rice leaf morphogenesis, especially in the formation of bulliform cells. Bulliform cells are a special type of bubble‐shaped cells between two vascular bundles in the adaxial side of leaves in gramineous plants and generally occur in longitudinal strips. Based on the difference in the number and size of bulliform cells and their adjacent epidermal cells observed in the mutant lines with increased or decreased expression level of *ROC8*, it is convincing that, just like *ROC5* (Zou *et al*., 2011), *ROC8* also regulates both the number and size of bulliform cells, not just the size of bulliform cells as previously reported (Sun *et al*., 2020).

Overexpression and knockout of *ROC8* had similar mutant phenotypes as that of *ROC5*, suggesting that they are not functionally redundant, instead, may function cooperatively. We therefore investigated their interaction and compared their homodimerization and heterodimerization to know whether they function in partnership. Our results revealed that both ROC8 and ROC5 could individually form homodimers (Figure 2), and that ROC8 could interact with ROC5 to form heterodimer (Figure 3; Figure S6), providing evidence for the speculation that ROC members could form homodimer and heterodimer in planta (Ito *et al*., 2003). Notably, we demonstrated that the ROC8‐ROC5 heterodimer rather than the homodimer of ROC8 or ROC5 was functional in regulating leaf rolling, as neither overexpression of *ROC8* in the *oul1* mutant (*roc5*) nor overexpression of *ROC5* in the *roc8* mutant line crispr8‐6 could rescue the mutant phenotype of abaxial rolling leaf (Figure 5; Figure 6). This finding explains why mutation in *ROC8* or *ROC5* leads to similar mutant phenotypes. In addition, the function of *ROC8* and *ROC5* seems to be additive as their double mutants showed stronger abaxially rolled leaves than the single mutant (Figure S7).

Many leaf‐rolling genes have been reported to regulate the number, size, and arrangement of bulliform cells by regulating the formation and composition of secondary cell wall (Fang *et al*., 2012; Hu *et al*., 2010; Yang *et al*., 2014), and the function of vacuoles via water homeostasis (Li *et al*., 2017; Xiang *et al*., 2012). Some leaf‐rolling genes, such as *SLL1* and *SRL2,* have also been shown to affect leaf rolling by regulating differentiation and development of mesophyll cells, sclerenchyma cells, and epidermal cells (Liu *et al*., 2016; Zhang *et al*., 2009). Moreover, genes involved in auxin biosynthesis can also affect the water content in bulliform cells to modulate rice leaf rolling (Fujino *et al*., 2008; Woo *et al*., 2007). Based on analysis of the hundreds of genes that were commonly differentially expressed in the *roc5roc8* double mutant, the *roc8* single mutant, and *oul1* (the *roc5* single mutant), it is evident that majority of these shared DEGs are related to metabolic process and cellular process (Figure 7, Data S4), consistent with the notion that regulation of the number and size of bulliform cells involves coordination of metabolic and cellular processes associated with cell differentiation and development, cell wall biosynthesis and formation, and vacuole functionality.

On the basis of the results generated by previous studies (Sun *et al*., 2020; Zou *et al*., 2011) and this study, we proposed a working model (Figure S8) to illustrate the competitive relationship between the homodimerization of ROC5 or ROC8 and the heterodimerization of ROC5 and ROC8, and the biological processes that are regulated by the ROC5‐ROC8 heterodimer and associated with leaf rolling. Overexpressing either *ROC8* or *ROC5* is capable of enhancing the heterodimerization of ROC8 and ROC5, due to the stronger and/or more stable heterodimers than homodimers (Figure 4), to repress the corresponding metabolic and cellular processes involved in inducing adaxial rolling of leaves. Knockout of *ROC8* or *ROC5* can dissociate the heterodimer of ROC8 and ROC5 to enhance the corresponding metabolic and cellular processes involved in inducing leaf rolling in the opposite direction, that is, abaxial rolling. We envision that maintaining a certain level of the ROC5‐ROC8 heterodimer is critical for normal leaf morphogenesis and development, which is achieved through the competitive relationship between the heterodimerization of ROC5‐ROC8 and the homodimerization of ROC5 or ROC8. How the competitive relationship is regulated is still an open question.

In conclusion, we demonstrated that the development and morphogenesis of bulliform cells of rice leaves, and consequently leaf rolling, are regulated by the ROC8‐ROC5 heterodimer rather than the homodimer of ROC8 or ROC5, although the downstream targets of the ROC8‐ROC5 heterodimer that are directly involved in division and expansion of bulliform cells are yet to be identified.

## Experimental procedures

### Plant materials, growth conditions, and phenotyping

The plant materials used in this study were *japonica* rice accession Kitaake, the *oul1* mutant (with the Nipponbare background), and transgenic plants related to these two accessions. All plants were grown in a greenhouse at the Jiangsu Academy of Agricultural Sciences, Nanjing, China. Leaf adaxial/abaxial‐rolling index was measured as previously described (Shi *et al*., 2007) with at least 30 individual plants.

### qRT‐PCR

For analysis of the expression levels of *ROC5* and *ROC8* in the wild‐type plant, the *oul1* mutant, transgenic lines, and various rice varieties, total RNA was extracted from the third leaves using a plant RNA extraction kit (Tiangen Co., Nanjing, China) according to the manufacturer's instructions. Each RNA sample was extracted from a pool of tissues collected from at least three individuals. For each sample, 1 μg of total RNA was reverse transcribed to cDNA using a reverse transcription kit (SuperScript II; TaKaRa). qRT‐PCR was performed using a SYBR Premix Ex TaqTM kit (TaKaRa) on an ABI prism 7500 Real‐Time PCR System. The rice *ubiquitin* gene (*Os03g0234200*) was used as a reference gene with the primer pair Ubq. All primer sequences are listed in Table S3.

### Yeast two‐hybrid assay

Fragments including different domains of ROC8, i.e., amino acids 1‐710 (F), 1‐200 (P1), 1‐70 (P2), 18‐70 (P3), 71‐710 (P4), 201‐710 (P5), 201‐435 (P6), 436‐710 (P7), and 71‐200 (P8) were PCR‐amplified and fused to the GAL4 binding domain in the pGBKT7 vector. Similar constructs were made for ROC5 using fragments including amino acids 1‐804 (F), 1‐308 (X1), 1‐153 (X2), 97‐153 (X3), 154‐804 (X4), 309‐804 (X5), 309‐544 (X6), 545‐804 (X7), 154‐308 (X8), 97‐308 (X9), and 154‐544 (X10). For both proteins, the PGBKT7 vector without fused fragment was used as control. In addition, the full‐length coding sequence of *ROC8* and *ROC5* was individually fused to the AD in the pGADT7 vector. The constructs were transformed into the yeast strain AH109 according to the manufacturer's instructions. The growth state of each transformant was examined on the SD/‐Trp‐His/‐Ade medium for the transcription activation activity assay, and on the SD/‐Trp/‐Leu/‐His/‐Ade medium for the protein interaction analysis. All the primer pairs used in amplifying the corresponding fragments are listed in Table S3.

### BiFC assay

The coding sequences of *ROC8* and *ROC5* were cloned into the binary BiFC vectors p2YN and p2YC to generated ROC8‐nYFP, ROC8‐cYFP, ROC5‐nYFP, and ROC5‐cYFP constructs, respectively. The coding sequences of *ROC8* and *ROC5* were also cloned into the pCAMBIA1305‐YFP vector (generated by insertion of a 35S promoter and an YFP expression cassette into the pCAMBIA1305 vector) to generate the ROC8‐YFP and ROC5‐YFP constructs, respectively, which were used as the positive controls. The fragments encoding amino acids 201‐435 (P6) of ROC8 and amino acids 545‐804 (X7) of ROC5 were cloned into the pCAMBIA1305‐35S vector (generated by deletion of the YFP expression cassette from the pCAMBIA1305‐YFP vector) to generate the 35S:P6 and 35S:X7 constructs, respectively.

For transient expression, the *Agrobacterium tumefaciens* strain EHA105 carrying the BiFC construct was co‐infiltrated into *N*. *benthamiana* leaves with the p19 strain and the nuclear marker, D53‐mCherry fusion protein (Zhou *et al*., 2013). Infiltrated leaves were observed 48 to 72 h after infiltration using a laser scanning confocal microscope (ZEISS LSM 700). The eYFP and mCherry fluorescent signals from the expressed fusion constructs were monitored sequentially. The excitation wavelengths for eYFP and mCherry are 514 and 587 nm, respectively, and the detection wavelengths for eYFP and mCherry are 527 and 610 nm, respectively. All primer sequences used in plasmid construction are listed in Table S3.

### Vector construction and rice transformation

For overexpression of *ROC8* in wild‐type rice Kitaake, the full‐length CDS of *ROC8* was amplified from Kitaake and then cloned into the pCAMBIA1305‐GFP vector (generated by insertion of a 35S promoter and a GFP expression cassette into the pCAMBIA1305 vector) to generate the ROC8‐GFP construct. Subsequently, the plasmid was introduced into the *Agrobacterium tumefaciens* strain EHA105 and used to infect the calli of Kitaake. The ROC8‐GFP construct was also used in Co‐IP assays.

To generate knockout lines of *ROC8*, a 20‐bp sequence specific to *ROC8* was synthesized and cloned into the entry vector pOs‐sgRNA, and then cloned into the gateway destination vector pOs‐Cas9. The resulting plasmid was introduced into Kitaake. Positive lines were confirmed by PCR followed by sequencing.

For overexpression of *ROC8* in the *oul1* mutant, the full‐length CDS of *ROC8* was amplified and then cloned into the pCAMBIA2300‐Actin1‐ocs vector to generate the 2300‐ROC8 construct. Subsequently, the plasmid was introduced into the *oul1* mutant.

For overexpression of *ROC5* in the *ROC8*‐knockout line crispr8‐6, the full‐length CDS of *ROC5* was amplified and then cloned into the pCAMBIA2300‐Actin1‐ocs vector to generate the 2300‐ROC5 construct. Subsequently, the plasmid was introduced into the mutant line crispr8‐6.

To generate *roc5roc8* double mutants, a 20‐bp sequence specific for *ROC5* or *ROC8* was synthesized and cloned into the entry vector pOsU3 and pOsU6a to generate the pOsU3‐ROC5 and pOsU6a‐ROC8 constructs, respectively. Then, pOsU3‐ROC5 and pOsU6a‐ROC8 were simultaneously cloned into the gateway destination vector pOs‐Cas9. The resulting plasmid was introduced into Kitaake. Positive lines were confirmed by PCR followed by sequencing. The primers and the 20‐bp sequences used in constructing these vectors are listed in Table S3.

### Co‐IP assays

The full‐length CDS of *ROC5* was amplified and then cloned into the pCAMBIA1305‐GFP vector to generate the ROC5‐GFP construct. The full‐length CDS of *ROC8* was cloned into the pCAMBIA1300‐221‐Flag vector (generated by insertion of a 35S promoter and a Flag tag into the pCAMBIA1300 vector) to generate the ROC8‐Flag construct. The full‐length CDS of *ROC5* was fused with His‐tags and then cloned into the pCAMBIA1305‐35S vector to generate the ROC5‐His construct.

Protoplasts isolated from wild‐type plants were transfected with desired plasmids and incubated overnight. Co‐IP assay was conducted as previously described (Liang *et al*., 2018). Total protein was extracted with protein extraction buffer (150mM KCl, 50mM HEPES [pH7.5], 0.4% Triton‐X 100, 1mM DTT, and proteinase inhibitor cocktail) and was incubated with GFP‐Trap magnetic beads (ChromoTek) for 2 h at 4°C with shaking. After washing with IP buffer, the IP samples were eluted in a reducing buffer, followed by SDS‐PAGE and immunoblot analyses using anti‐GFP (11814460001, Roche), anti‐Flag (F1804, Sigma‐Aldrich), anti‐His (D291‐7, MBL) antibodies. About 1% total volume was used as the loading volume. All primer sequences used in plasmid construction are listed in Table S3.

### RNA‐seq analysis

Total RNA was isolated from the third leaves using the TRIzol reagent. Fresh samples from three individuals were pooled to represent a single biological replicate and three biological replicates were used per genotype. The RNA samples were then sent to Genepioneer Biotechnologies Company (China) for RNA quantification and qualification, library construction, and transcriptome sequencing. Expression of genes was calculated using StringTie (Pertea *et al*., 2015). The DESeq2 package (Love *et al*., 2014) was used to identify the DEGs with the thresholds of false discovery rate (FDR) ≤ 0.05 and |log_2_ ratio| ≥ 1.

### Histological and microscopic examination

The basal half of each third leaf was collected at the vegetative stage and fixed in FAA. The samples were dehydrated through a graded series of ethanol, then embedded in Epon812 resin (Fluka) and polymerized. Sections (8 μm) were cut and stained with filtered 1% toluidine blue before microscopic examination (Leica DM5000B) and photographing.

The number of typical bulliform cells and the adjacent epidermal cells between the two small vascular bundles and those abutting the large vascular bundles were all counted. Four leaves were counted for each line, with each leaf counted only once.

### Accession numbers

*ROC5* (*LOC_Os02g45250.1*) is available in the MSU Rice Genome Annotation Project Database. *ROC8* (*XM_015786612.2*/*Os06t0208100‐01*) is available in the NCBI/Rice Annotation Project (RAP) Database and Resource.

## Conflicts of interest

The authors have submitted a patent application based on the results reported in this paper.

## Author contribution

Y. X., Q‐H. Z. and J. Y. designed the research; Y. X., W. K., F. W., J. W., Y. T., W. L., Z. C., F. F. and Y. J. performed the research; and Y. X. and Q‐H. Z. wrote the paper.

## Supporting information


**Figure S1** Characterization of ROC8 transcript and protein.
**Figure S2** Yeast two‐hybrid assay of the protein truncations without transcriptional activation activity.
**Figure S3** *ROC8* overexpression and knockout analyses.
**Figure S4** qRT‐PCR analysis of *ROC8* expression in the third leaf of the wild type Kitaake and three independent *ROC8*‐knockout lines crispr8‐6, crispr8‐7, and crispr8‐8.
**Figure S5** qRT‐PCR analysis of *ROC5* and *ROC8*.
**Figure S6** Yeast two‐hybrid assay.
**Figure S7** Characterization of *roc5roc8* double mutants generated by Crispr/Cas9‐mediated gene editing.
**Figure S8** A proposed working model for ROC‐mediated regulation of leaf rolling.
**Table S1** Sequencing results of the T_0_ *roc5roc8* double mutant lines generated by gene editing.
**Table S2** Differentially expressed genes associated with lignin biosynthetic process, cell wall and vacuole formation, and water stress in leaves.
**Table S3** List of primers used in this study.

**Data S1** Genes differentially expressed between the double mutant line crispr5&8‐1 and Kitaake.

**Data S2** Genes differentially expressed between the single mutant line crispr8‐6 and Kitaake.

**Data S3** Genes differentially expressed between the *oul1* mutant and Nipponbare.

**Data S4** Differentially expressed genes shared among the double mutant line crispr5&8‐1, the single mutant line crispr8‐6, and *oul1*.
