# Supplementary material for: Heterodimer formed by ROC8 and ROC5 modulates leaf rolling in rice
Source: Plant Biotechnol J. 2021 Sep 8;19(12):2662–72. doi: 10.1111/pbi.13690 (PMC8633501; doi:10.1111/pbi.13690)
Supplement: Supplementary file 1 — Figure S1 Characterization of ROC8 transcript and protein. Figure S2 Yeast two‐hybrid assay of the protein truncations without transcriptional activation activity. Figure S3 ROC8 overexpression and knockout analyses. Figure S4 qRT‐PCR analysis of ROC8 expression in the third leaf of the wild type Kitaake and three independent ROC8‐knockout lines crispr8‐6, crispr8‐7, and crispr8‐8. Figure S5 qRT‐PCR analysis of ROC5 and ROC8. Figure S6 Yeast two‐hybrid assay. Figure S7 Characterization of roc5roc8 double mutants generated by Crispr/Cas9‐mediated gene editing. Figure S8 A proposed working model for ROC‐mediated regulation of leaf rolling. Table S1 Sequencing results of the T0 roc5roc8 double mutant lines generated by gene editing. Table S2 Differentially expressed genes associated with lignin biosynthetic process, cell wall and vacuole formation, and water stress in leaves. Table S3 List of primers used in this study. [file PBI-19-2662-s001.docx]

**SUPPLEMENTAL FIGURES:**


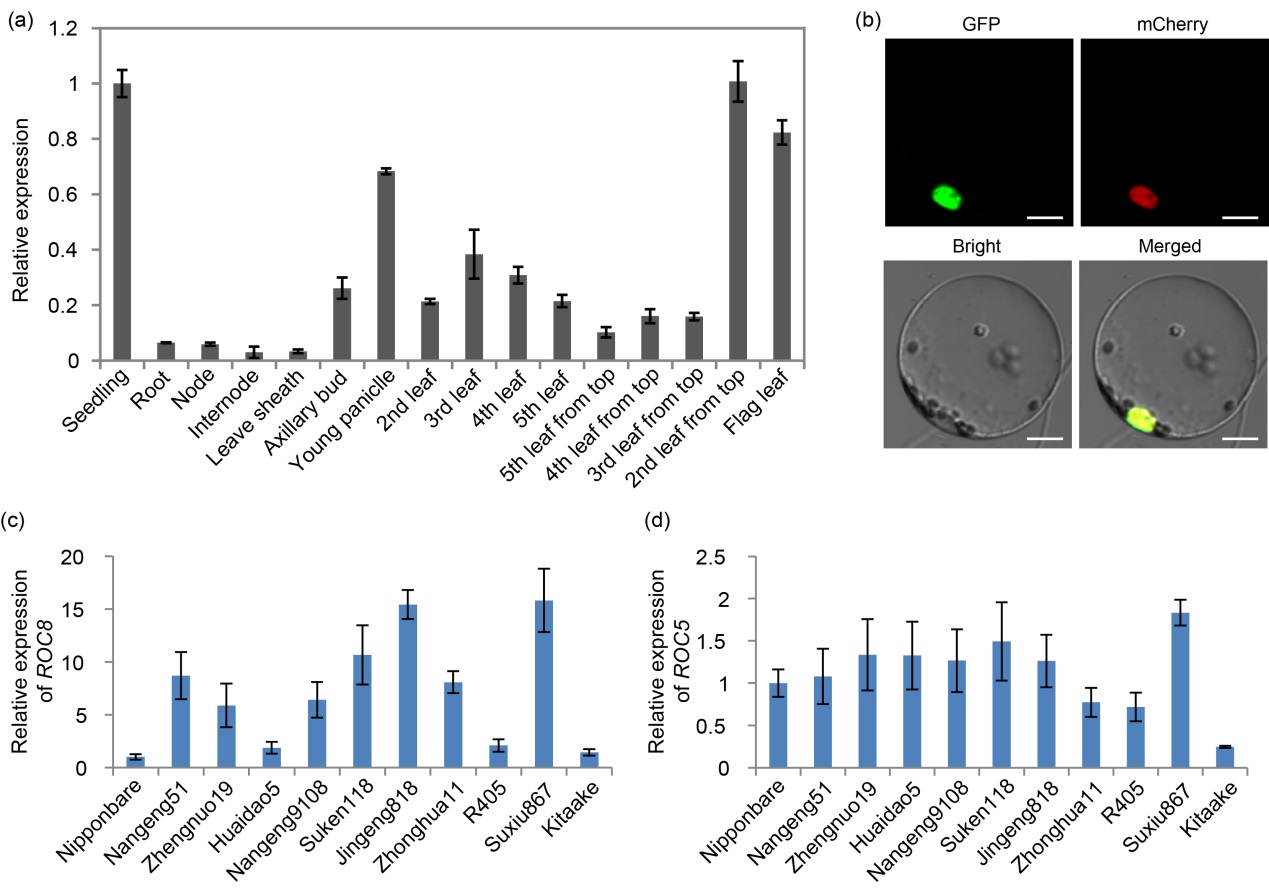


**Figure S1** Characterization of ROC8 transcript and protein. (a) qRT-PCR analysis of *ROC8* expression in different organs in wild type Kitaake. Data are means ± SD (n = 3). (b) Subcellular localization of ROC8 in rice protoplasts. GFP, fluorescence of ROC8-GFP fusion protein, mCherry, fluorescence of nuclear marker D53-mCherry fusion protein; Bright, bright-field; Merged, merged image of GFP, mCherry, and Bright. Bars:10 μm. (c, d) qRT-PCR analysis of *ROC8* (c) and *ROC5* (d) expression in the third leaf of rice variety Nipponbare, Nangeng51, Zhengnuo19, Huaidao5, Nangeng9108, Suken118, Jingeng818, Zhonghua11, R405, Suxiu867, and Kitaake. Data are means ± SD (n = 3).


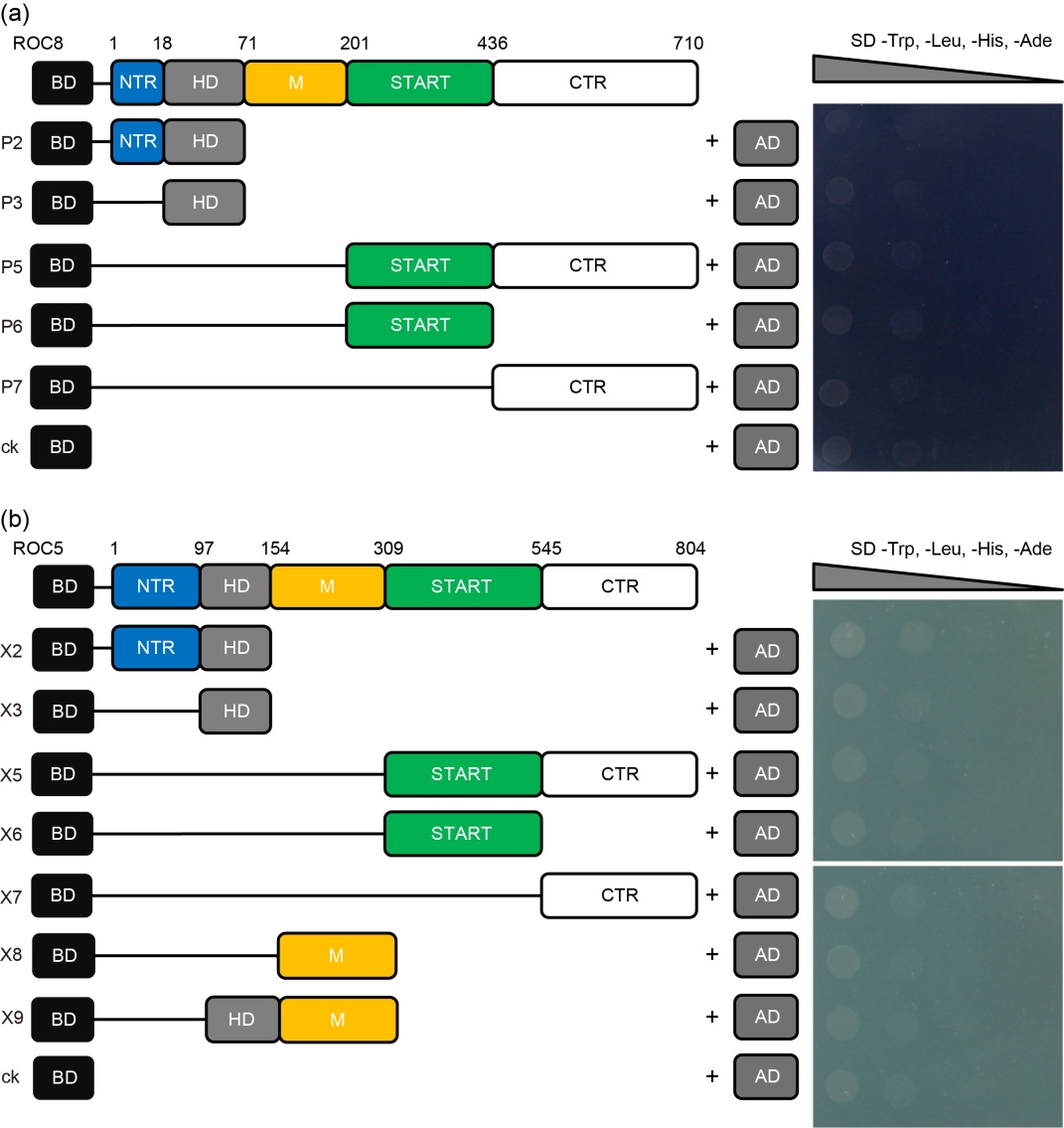


**Figure S2** Yeast two-hybrid assay of the protein truncations without transcriptional activation activity. Numbers indicate the amino acid position. P2, P3, P5, P6, and P7 indicate a series of truncated ROC8 proteins without transcriptional activation activity fused with the BD (a). X2, X3, X5, X6, X7, X8, and X9 indicate a series of truncated ROC5 proteins without transcriptional activation activity fused with the BD (b). The empty pGBKT7 co-transformed with the empty pGADT7 was used as a negative control (ck).


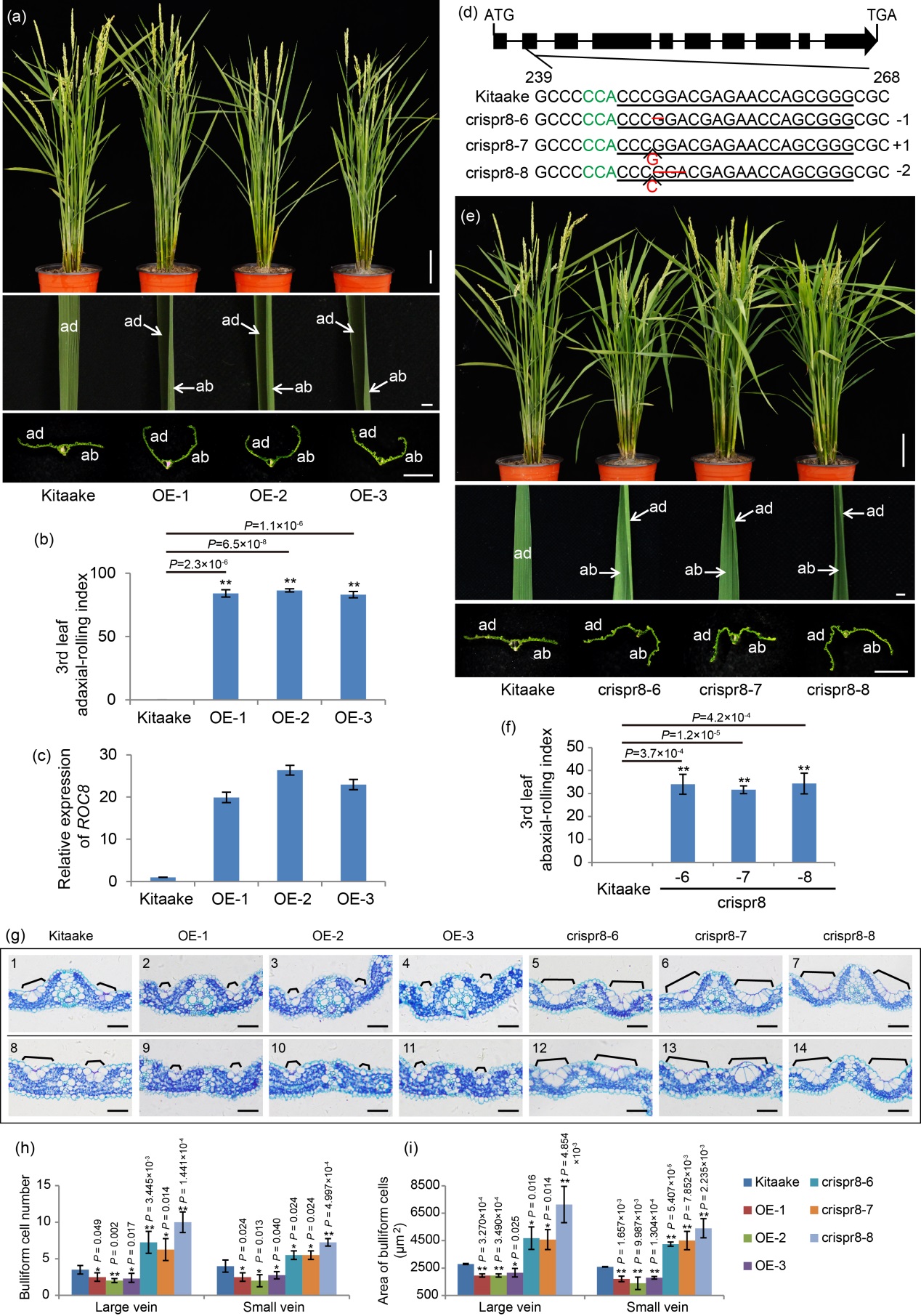


**Figure S3** *ROC8* overexpression and knockout analyses. (a) Plant stature (top), leaf phenotype (middle), and transverse leaf sections (bottom) of the wild type Kitaake and three independent *ROC8* overexpression lines OE-1, OE-2, and OE-3 at the heading stage, showing that the leaves of the overexpression lines were adaxially rolled. ab, abaxial; ad, adaxial. Bars: 10 cm (top) and 5 mm (middle and bottom). (b) Leaf adaxial-rolling index of the third leaf. Data are means ± SD (n = 30). **P<0.01 by the Student's *t* test. (c) qRT-PCR analysis of *ROC8* expression. Data are means ± SD (n = 3). (d) Sketch map of the Crispr/Cas9-induced mutations in *ROC8* in three independent knockout lines crispr8-6, crispr8-7, and crispr8-8. PAM motifs are shown in green letters. The 20-bp gene-specific sequences are underlined. The number of nucleotides deleted (-) or inserted (+) is shown on the right of the figure. (e) Plant stature (top), leaf phenotype (middle), and transverse leaf sections (bottom) of the wild type Kitaake and three independent *ROC8* knockout lines at the heading stage, showing that the leaves of knockout lines were abaxially rolled. ab, abaxial; ad, adaxial. Bars: 10 cm (top) and 5 mm (middle and bottom). (f) Leaf abaxial-rolling index of the third leaf. Data are means ± SD (n = 30). **P<0.01 by the Student's *t* test. (g) Cross sections showing the bulliform cells abutting large vascular (panels 1 to 7) and those between the two small vascular bundles (panels 8 to 14) in the wild type Kitaake, three *ROC8* overexpression lines, and three *ROC8* knockout lines. Black bracket lines indicate the bulliform cells. Bars: 50 μm. (h, i) Statistical analysis of the number (h) and area (i) of bulliform cells. Data are means ± SD (n = 4). *P<0.05 and **P<0.01 by the Student's *t* test.


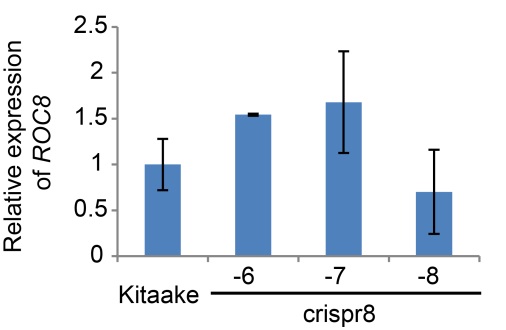


**Figure S4** qRT-PCR analysis of *ROC8* expression in the third leaf of the wild type Kitaake and three independent *ROC8*-knockout lines crispr8-6, crispr8-7, and crispr8-8. Data are means ± SD (n = 3).


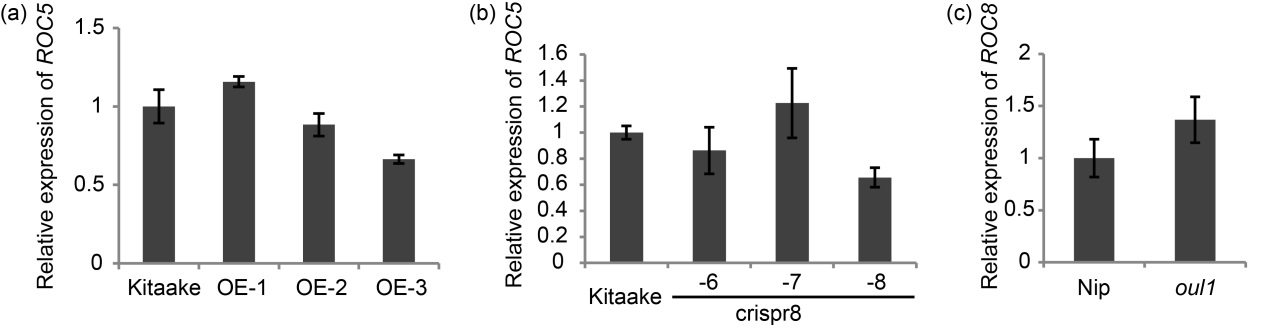


**Figure S5** qRT-PCR analysis of *ROC5* and *ROC8*. (a) qRT-PCR analysis of *ROC5* expression in the third leaf of the wild type Kitaake and three independent *ROC8* overexpression lines OE-1, OE-2, and OE-3. (b) qRT-PCR analysis of *ROC5* expression in the third leaf of Kitaake and three independent *ROC8*-knockout lines crispr8-6, crispr8-7, and crispr8-8. (c) qRT-PCR analysis of *ROC8* expression in the third leaf of Nipponbare (Nip) and the mutant *oul1*. Data are means ± SD (n = 3).


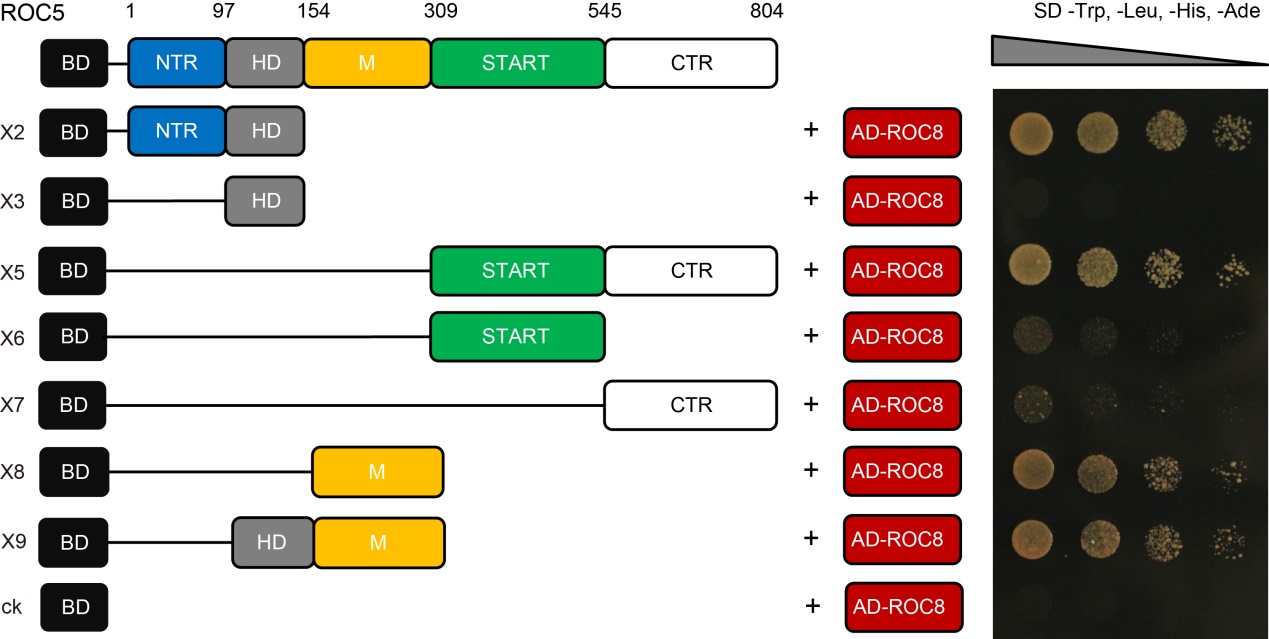


**Figure S6** Yeast two-hybrid assay. Numbers indicate the amino acid position. X2, X3, X5, X6, X7, X8, and X9 indicate a series of truncated ROC5 proteins without transcriptional activation activity fused with the BD. The empty pGBKT7 co-transformed with the ROC8 fused with the AD was used as a negative control.


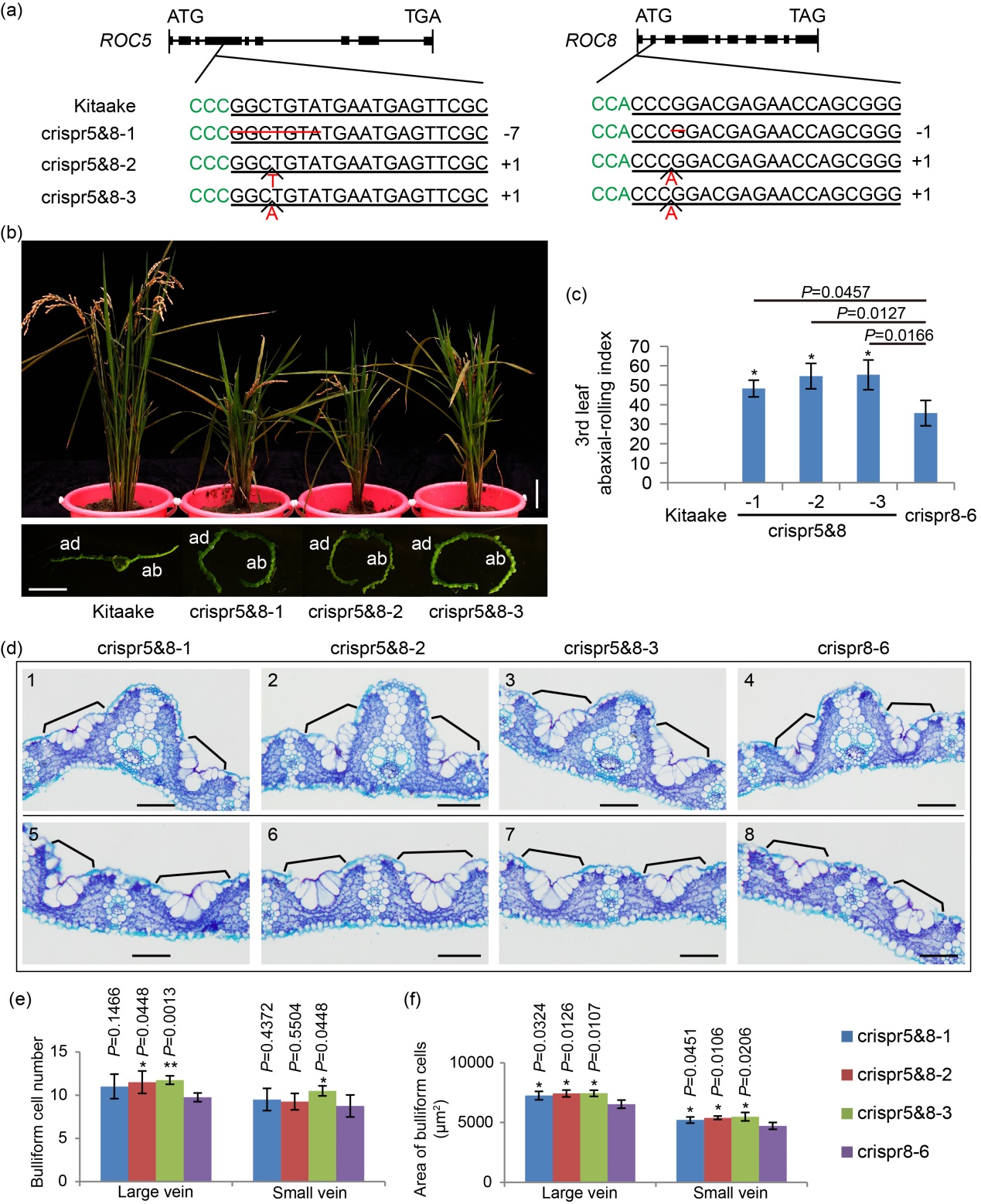


**Figure S7** Characterization of *roc5roc8* double mutants generated by Crispr/Cas9-mediated gene editing. (a) Sketch map of the mutations in *ROC5* (left panel) and *ROC8* (right panel) in the knockout lines. The deleted and inserted nucleotides are indicated by red bars and red upper-case letters, respectively. PAM motifs are shown in green letters. The 20-bp gene-specific sequences are underlined. The number of nucleotides deleted (-) or inserted (+) is shown on the right. (b) Plant stature (top) and transverse leaf sections (bottom) of wild type Kitaake and three independent double mutant lines crispr5&8-1, crispr5&8-2, and crispr5&8-3 at the heading stage. Note that the leaves of the mutant lines were significantly abaxially rolled. ab, abaxial; ad, adaxial. Bars: 10 cm (top) and 5 mm (bottom). (c) Leaf abaxial-rolling index of the third leaf. Data are means ± SD (n = 30). (d) Cross sections showing the bulliform cells abutting large vascular (panels 1 to 4) and those between the two small vascular bundles (panels 5 to 8) in the single mutant line crispr8-6 and three double mutant lines. Black bracket lines indicate the bulliform cells. Bars: 50 μm. (e, f) Statistical analysis of the number (e) and area (f) of bulliform cells. Data are means ± SD (n = 4). *P<0.05 and **P<0.01 by the Student's *t* test.


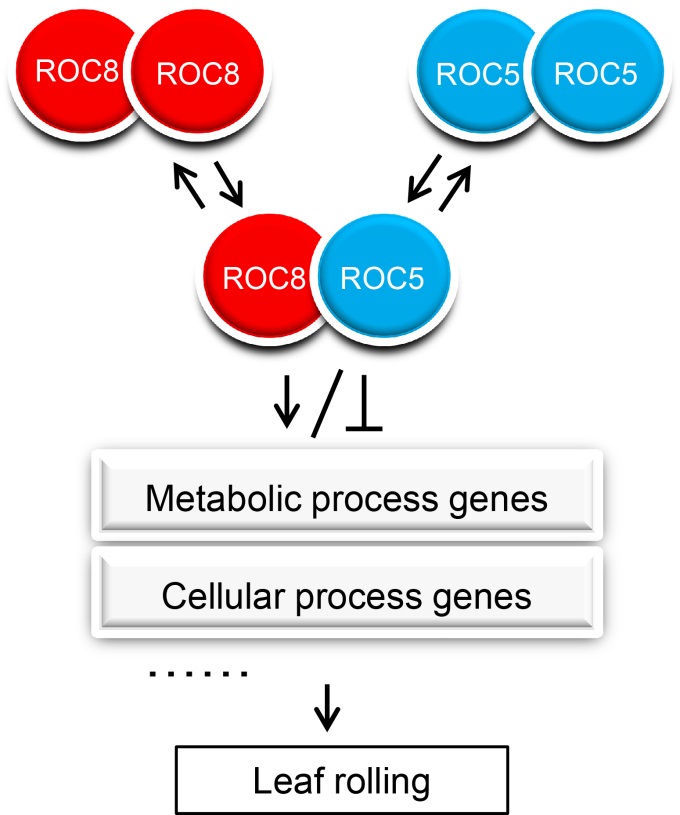


**Figure S8** A proposed working model for ROC-mediated regulation of leaf rolling. ROC-mediated regulation of leaf rolling is achieved through regulation of genes involved in metabolic and cellular processes by the heterodimer formed by interaction between ROC8 and ROC5. There is a competitive relationship between the formation of the ROC8-ROC5 heterodimer and the formation of the ROC8-ROC8 homodimer or the ROC5-ROC5 homodimer.

Table S1 Sequencing results of the T_0_ *roc5roc8* double mutant lines generated by gene editing.

| Line | Target site of *ROC5* | Target site of *ROC8* | Genotype |
| --- | --- | --- | --- |
| Kitaake | CCCGGCTGTATGAATGAGTTCGC | CCACCCGGACGAGAACCAGCGGG | Wild type |
| crispr5&8-1 | Allele1: CCC---------------TGAATGAGTTCGC (-7)  Allele2: CCC---------------TGAATGAGTTCGC (-7) | Allele1: CCACCC--GACGAGAACCAGCGGG (-1)  Allele2: CCACCC--GACGAGAACCAGCGGG (-1) | Homozygous |
| crispr5&8-2 | Allele1: CCCGGCTTGTATGAATGAGTTCGC (+1)  Allele2: CCCGGCTTGTATGAATGAGTTCGC (+1) | Allele1: CCACCCAGGACGAGAACCAGCGGG (+1)  Allele2: CCACCCAGGACGAGAACCAGCGGG (+1) | Homozygous |
| crispr5&8-3 | Allele1: CCCGGCATGTATGAATGAGTTCGC (+1)  Allele2: CCCGGCATGTATGAATGAGTTCGC (+1) | Allele1: CCACCCAGGACGAGAACCAGCGGG (+1)  Allele2: CCACCCAGGACGAGAACCAGCGGG (+1) | Homozygous |
| crispr5&8-4 | Allele1: CCCGGC--GTATGAATGAGTTCGC (-1)  Allele2: CCCGGC----TATGAATGAGTTCGC (-2) | Allele1: CCACCCAGGACGAGAACCAGCGGG (+1)  Allele2: CCACCCAGGACGAGAACCAGCGGG (+1) | Biallelic |
| crispr5&8-5 | Allele1: CCCGGCT-------GAATGAGTTCGC (-4)  Allele2: CCCGGCT-------GAATGAGTTCGC (-4) | Allele1: CCACCCTGGACGAGAACCAGCGGG (+1)  Allele2: CCACCCTGGACGAGAACCAGCGGG (+1) | Homozygous |
| crispr5&8-6 | Allele1: CCCGGCTTGTATGAATGAGTTCGC (+1)  Allele2: CCCGGCATGTATGAATGAGTTCGC (+1) | Allele1: CCACCCTGGACGAGAACCAGCGGG (+1)  Allele2: CCACCCAGGACGAGAACCAGCGGG (+1) | Biallelic |
| crispr5&8-7 | Allele1: CCCGGCT-------GAATGAGTTCGC (-4)  Allele2: CCCGGCTTGTATGAATGAGTTCGC (+1) | Allele1: CCACCC--GACGAGAACCAGCGGG (-1)  Allele2: CCACCCAGGACGAGAACCAGCGGG (+1) | Biallelic |

PAM motifs are shown in green letters; The 20-bp gene-specific sequences are underlined; Insertion sequence is shown in red letter; Deletion sequence is shown by dashed line; Minus (-) and plus (+) signs indicate the number of nucleotides deleted and inserted, respectively.

Table S2 Differentially expressed genes associated with lignin biosynthetic process, cell wall and vacuole formation, and water stress in leaves

| #Gene_ID | Gene Ontology annotation | Regulation |
| --- | --- | --- |
| LOC_Os04g15920 | Biological Process: lignin biosynthetic process (GO:0009809) | Up |
| LOC_Os06g48180 | Biological Process: cell wall modification (GO:0042545) | Up |
| LOC_Os02g17600 | Biological Process: cell wall biogenesis (GO:0042546) | Down |
| LOC_Os02g17590 | Biological Process: cell wall biogenesis (GO:0042546) | Down |
| MSTRG.3717 | Biological Process: cell wall organization (GO:0071555) | Down |
| LOC_Os06g51050 | Biological Process: cell wall macromolecule catabolic process (GO:0016998) | Up |
| LOC_Os05g01140 | Biological Process: response to water deprivation (GO:0009414) | Up |
| LOC_Os05g18780 | Cellular Component: vacuole (GO:0005773) | Up |
| LOC_Os01g52130 | Molecular Function: secondary active sulfate transmembrane transporter activity (GO:0008271) | Up |
| LOC_Os12g01370 | Molecular Function: oxidoreductase activity, acting on paired donors, with oxidation of a pair of donors resulting in the reduction of molecular oxygen to two molecules of water (GO:0016717) | Up |

Table S3. List of primers used in this study

| Gene cloning primer | | | | | | | | | |
| --- | --- | --- | --- | --- | --- | --- | --- | --- | --- |
| Primer name | Forward sequence (5’-3’) | | | | Reverse sequence (5’-3’) | | | | |
| ROC8 | ATGGATTTCGGCGACGAACC | | | | TCAGGGGTGGTGGCCATGGG | | | | |
| Quantitative PCR Primers | | | | | | | | | |
| Primer name | Forward sequence (5’-3’) | | | | Reverse sequence (5’-3’) | | | | |
| qRT-ROC8 | GCTCCAACGAGGTGAGCG | | | | GGACTTGATTGCCGTGCGA | | | | |
| qRT-ROC5 | CGCAAGAGGAAGAAGCGATAC | | | | GCTCCAGTTGCGTCTTCATC | | | | |
| Ubq | GCTCCGTGGCGGTATCAT | | | | CGGCAGTTGACAGCCCTAG | | | | |
| Binary vector construction primers | | | | | | | | | |
| Primer name | | | | Primer sequence (5' to 3') | | Restriction enzyme | | | |
| ROC8-1305GFP-SpeI-F | | ATT**ACTAGT**ATGGATTTCGGCGACGAACC | | | | | | SpeI | |
| ROC8-1305GFP-BamHI-R | | AAT**GGATCC**GGGGTGGTGGCCATGGG | | | | | | BamHI | |
| ROC5-1305GFP-SpeI-F | | ATT**ACTAGT**ATGAGCTTTGGGGGCCT | | | | | | SpeI | |
| ROC5-1305GFP-BamHI-R | | AAT**GGATCC**GGCGTCGCACTGCAGCGCC | | | | | | BamHI | |
| ROC8-1300Flag-XbaI-F | | CACGGGGGAC**TCTAGA**ATGGATTTCGGCGACGAACC | | | | | | XbaI | |
| ROC8-1300Flag-SalI-R | | TGTAGTCCAT**GTCGAC**GGGGTGGTGGCCATGGGCGG | | | | | | SalI | |
| ROC5-1305His-SpeI-F | | GCCCAGATCA**ACTAGT**ATGAGCTTTGGGGGCCTCTT | | | | | | SpeI | |
| ROC5-1305His-BglII-R | | GGCCGCTTTA**AGATCT**TCAGTGGTGGTGGTGGTGGTGGGCGTCGCACTGCAGCGCCG | | | | | | BglII | |
| ROC8-2300-SmaI-F | | GTAGAAGAGGTA**CCCGGG**ATGAGCTTTGGGGGCCTCTT | | | | | | SmaI | |
| ROC8-2300-SmaI-R | | CTCTAGAGGATC**CCCGGG**TCAGGCGTCGCACTGCAGCG | | | | | | SmaI | |
| ROC5-2300-SmaI-F | | GTAGAAGAGGTA**CCCGGG**ATGGATTTCGGCGACGAACC | | | | | | SmaI | |
| ROC5-2300-SmaI-R | | CTCTAGAGGATC**CCCGGG**TCAGGGGTGGTGGCCATGGG | | | | | | SmaI | |
| ROC8-pOs-Cas9-F^#^ | | AGATGATCCGTGGCACCCGCTGGTTCTCGTCCGGGGTTTTAGAGCTATGC | | | | | | BsaI | |
| ROC8-pOs-Cas9-R | | GCATAGCTCTAAAACCCCGGACGAGAACCAGCGGGTGCCACGGATCATCT | | | | | | BsaI | |
| ROC5-U3-F | | ggcaGCGAACTCATTCATACAGCC | | | | | | BsaI | |
| ROC5-U3-R | | aaacGGCTGTATGAATGAGTTCGC | | | | | | BsaI | |
| ROC8-U6a-F | | gccgCCCGCTGGTTCTCGTCCGGG | | | | | | BsaI | |
| ROC8-U6a-R | | aaacCCCGGACGAGAACCAGCGGG | | | | | | BsaI | |
| Subcellular localization in rice protoplasts primer | | | | | | | | | |
| Primer name | | Primer sequence (5' to 3') | | | | | Restriction enzyme | | |
| ROC8-pA7-GFP-XhoI-F | | | ACGAACGATA**CTCGAG**ATGGATTTCGGCGACGAACC | | | | | XhoI | |
| ROC8-pA7-GFP-SpeI-R | | | GCTCACCATC**ACTAGT**GGGGTGGTGGCCATGGG | | | | | SpeI | |
| Yeast two-hybrid vector construction primers | | | | | | | | | |
| Primer name | | Primer sequence (5' to 3') | | | | | Restriction enzyme | | |
| ROC8-pGADT7-EcoRI-F | | ATGGAGGCCAGT**GAATTC**ATGGATTTCGGCGACGAACC | | | | | | EcoRI | |
| ROC8-pGADT7-EcoRI-R | | CCCACCCGGGTG**GAATTC**GGGGTGGTGGCCATGGG | | | | | | EcoRI | |
| ROC5-pGADT7-EcoRI-F | | ATGGAGGCCAGT**GAATTC**ATGAGCTTTGGGGGCCT | | | | | | EcoRI | |
| ROC5-pGADT7-EcoRI-R | | CCCACCCGGGTG**GAATTC**GGCGTCGCACTGCAGCGCC | | | | | | EcoRI | |
| ROC8-pGBKT7-EcoRI-F | | CATGGAGGCC**GAATTC**ATGGATTTCGGCGACGAACC | | | | | | EcoRI | |
| ROC8-pGBKT7-EcoRI-R | | GGATCCCCGG**GAATTC**GGGGTGGTGGCCATGGG | | | | | | EcoRI | |
| ROC5-pGBKT7-EcoRI-F | | CATGGAGGCC**GAATTC**ATGAGCTTTGGGGGCCT | | | | | | EcoRI | |
| ROC5-pGBKT7-EcoRI-R | | GGATCCCCGG**GAATTC**GGCGTCGCACTGCAGCGCC | | | | | | EcoRI | |
| P1-pGBKT7-EcoRI-F | | CATGGAGGCC**GAATTC**ATGAGCTTTGGGGGCCT | | | | | | EcoRI | |
| P1-pGBKT7-BamHI-R | | GCAGGTCGAC**GGATCC**CTCCATGTCGGACACGGGCG | | | | | | BamHI | |
| P2-pGBKT7-EcoRI-F | | CATGGAGGCC**GAATTC**ATGAGCTTTGGGGGCCT | | | | | | EcoRI | |
| P2-pGBKT7-BamHI-R | | GCAGGTCGAC**GGATCC**CTTCATCTGGGTCCGGCGAT | | | | | | BamHI | |
| P3-pGBKT7-EcoRI-F | | CATGGAGGCC**GAATTC**CGCTACCACCGCCACACGCC | | | | | | EcoRI | |
| P3-pGBKT7-BamHI-R | | GCAGGTCGAC**GGATCC**CTTCATCTGGGTCCGGCGAT | | | | | | BamHI | |
| P4-pGBKT7-EcoRI-F | | CATGGAGGCC**GAATTC**GCGCAGCACGAGCGGGCGGA | | | | | | EcoRI | |
| P4-pGBKT7-BamHI-R | | GCAGGTCGAC**GGATCC**GGGGTGGTGGCCATGGGCGG | | | | | | BamHI | |
| P5-pGBKT7-EcoRI-F | | CATGGAGGCC**GAATTC**CGGCCCATGATGGCCGAGAT | | | | | | EcoRI | |
| P5-pGBKT7-BamHI-R | | GCAGGTCGAC**GGATCC**GGGGTGGTGGCCATGGGCGG | | | | | | BamHI | |
| P6-pGBKT7-EcoRI-F | | CATGGAGGCC**GAATTC**CGGCCCATGATGGCCGAGAT | | | | | | EcoRI | |
| P6-pGBKT7-BamHI-R | | GCAGGTCGAC**GGATCC**CTCGCACGCGCGCTGGAGCG | | | | | | BamHI | |
| P7-pGBKT7-EcoRI-F | | CATGGAGGCC**GAATTC**CGCTACGCCTCCCTCGTCGC | | | | | | EcoRI | |
| P7-pGBKT7-BamHI-R | | GCAGGTCGAC**GGATCC**GGGGTGGTGGCCATGGGCGG | | | | | | BamHI | |
| P8-pGBKT7-EcoRI-F | | CATGGAGGCC**GAATTC**GCGCAGCACGAGCGGGCGGA | | | | | | EcoRI | |
| P8-pGBKT7-BamHI-R | | GCAGGTCGAC**GGATCC**CTCCATGTCGGACACGGGCG | | | | | | BamHI | |
| X1-pGBKT7-EcoRI-F | | CATGGAGGCC**GAATTC**ATGAGCTTTGGGGGCCTCTT | | | | | | EcoRI | |
| X1-pGBKT7-BamHI-R | | GCAGGTCGAC**GGATCC**GTCGATGTTACCCACCAGCG | | | | | | BamHI | |
| X2-pGBKT7-EcoRI-F | | CATGGAGGCC**GAATTC**ATGAGCTTTGGGGGCCTCTT | | | | | | EcoRI | |
| X2-pGBKT7-BamHI-R | | GCAGGTCGAC**GGATCC**CTTCATCTGCGTGCGGCGAT | | | | | | BamHI | |
| X3-pGBKT7-EcoRI-F | | CATGGAGGCC**GAATTC**AAGAGGAAGAAGCGATACCA | | | | | | EcoRI | |
| X3-pGBKT7-BamHI-R | | GCAGGTCGAC**GGATCC**CTTCATCTGCGTGCGGCGAT | | | | | | BamHI | |
| X4-pGBKT7-EcoRI-F | | CATGGAGGCC**GAATTC**ACGCAACTGGAGCGGCACGA | | | | | | EcoRI | |
| X4-pGBKT7-BamHI-R | | GCAGGTCGAC**GGATCC**GGCGTCGCACTGCAGCGCCG | | | | | | BamHI | |
| X5-pGBKT7-EcoRI-F | | CATGGAGGCC**GAATTC**AGGTCCGTGTTCTTAGAGCT | | | | | | EcoRI | |
| X5-pGBKT7-BamHI-R | | GCAGGTCGAC**GGATCC**GGCGTCGCACTGCAGCGCCG | | | | | | BamHI | |
| X6-pGBKT7-EcoRI-F | | CATGGAGGCC**GAATTC**AGGTCCGTGTTCTTAGAGCT | | | | | | EcoRI | |
| X6-pGBKT7-BamHI-R | | GCAGGTCGAC**GGATCC**TTCGCATTGGCGCTGCAGCG | | | | | | BamHI | |
| X7-pGBKT7-EcoRI-F | | CATGGAGGCC**GAATTC**TGCCTTGCCATCCTCATGTC | | | | | | EcoRI | |
| X7-pGBKT7-BamHI-R | | GCAGGTCGAC**GGATCC**GGCGTCGCACTGCAGCGCCG | | | | | | BamHI | |
| X8-pGBKT7-EcoRI-F | | CATGGAGGCC**GAATTC**ACGCAACTGGAGCGGCACGA | | | | | | EcoRI | |
| X8-pGBKT7-BamHI-R | | GCAGGTCGAC**GGATCC**GTCGATGTTACCCACCAGCG | | | | | | BamHI | |
| X9-pGBKT7-EcoRI-F | | CATGGAGGCC**GAATTC**AAGAGGAAGAAGCGATACCA | | | | | | EcoRI | |
| X9-pGBKT7-BamHI-R | | GCAGGTCGAC**GGATCC**GTCGATGTTACCCACCAGCG | | | | | | BamHI | |
| X10-pGBKT7-EcoRI-F | | CATGGAGGCC**GAATTC**ACGCAACTGGAGCGGCACGA | | | | | | EcoRI | |
| X10-pGBKT7-BamHI-R | | GCAGGTCGAC**GGATCC**TTCGCATTGGCGCTGCAGCG | | | | | | BamHI | |
| BiFC assay primers | | | | | | | | | |
| Primer name | | | | Primer sequence (5' to 3') | | | Restriction enzyme | | |
| ROC8-p2Y-PacI-F | | | | CATTTACGAACGATAG**TTAATTAA**ATGGATTTCGGCGACGAACC | | | | PacI | |
| ROC8-p2Y-SpeI-R | | | | CACTGCCACCTCCTCC**ACTAGT**GGGGTGGTGGCCATGGG | | | | SpeI | |
| ROC5-p2Y-PacI-F | | | | CATTTACGAACGATAG**TTAATTAA**ATGAGCTTTGGGGGCCT | | | | PacI | |
| ROC5-p2Y-SpeI-R | | | | CACTGCCACCTCCTCC**ACTAGT**GGCGTCGCACTGCAGCGCC | | | | SpeI | |
| ROC8-1305YFP-SpeI-F | | | | ATT**ACTAGT**ATGGATTTCGGCGACGAACC | | | | SpeI | |
| ROC8-1305YFP-BamHI-R | | | | AAT**GGATCC**GGGGTGGTGGCCATGGG | | | | BamHI | |
| ROC5-1305YFP-SpeI-F | | | | ATT**ACTAGT**ATGAGCTTTGGGGGCCT | | | | SpeI | |
| ROC5-1305YFP-BamHI-R | | | | AAT**GGATCC**GGCGTCGCACTGCAGCGCC | | | | BamHI | |
| P6-1305-35S-SpeI-F | | | | ATT**ACTAGT**CGGCCCATGATGGCCGAGAT | | | | | SpeI |
| P6-1305-35S-BglII-R | | | | AAT**AGATCT**CTCGCACGCGCGCTGGAGCG | | | | | BglII |
| X7-1305-35S-SpeI-F | | | | ATT**ACTAGT**TGCCTTGCCATCCTCATGTC | | | | | SpeI |
| X7-1305-35S-BglII-R | | | | AAT**AGATCT**GGCGTCGCACTGCAGCGCCG | | | | | BglII |

^#^ The 20-bp gene-specific sequences are underlined.
